# AHNAK2: a potential diagnostic biomarker for pancreatic cancer related to cellular motility

**DOI:** 10.1038/s41598-025-87337-5

**Published:** 2025-01-23

**Authors:** Mohamed Zardab, Barts Pancreas Tissue Bank, Rhiannon Roberts, Christine Hughes, Ahmet Imrali, Amina Saad, Claude Chelala, Richard P. Grose, Hemant M. Kocher

**Affiliations:** 1 Centre for Tumour Biology, Barts Cancer Institute, Queen Mary University of London, Charterhouse Square, London, EC1M 6BQ UK; 2 Barts and the London HPB Centre, The Royal London Hospital, Barts Health NHS Trust, London, UK

**Keywords:** Pancreatic ductal adenocarcinoma, Oncogene, Ezrin, Cortactin, Early diagnosis, Pancreatic cancer, Diagnostic markers

## Abstract

Pancreatic ductal adenocarcinoma lacks suitable biomarkers for early diagnosis of disease. In gene panels developed for early diagnosis of pancreatic cancer, high *AHNAK2* mRNA expression was one possible biomarker. In silico analysis of published human sample datasets (n = 177) and ex vivo analysis of human plasma samples (n = 30 PDAC with matched 30 healthy control) suggested AHNAK2 could be a diagnostic biomarker. At a plasma level of 421.47 ng/ml, AHNAK2 could potentially diagnose PDAC with a specificity and sensitivity of 83.33% and 86.67%. In vitro analysis suggests that in cell lines with diffuse cytoplasmic distribution of AHNAK2, there was colocalization of AHNAK2 with Cortactin in filipodia. This colocalization increased when cells were cultured on substrates such as Fibronectin and Collagen, as well as in hypoxia, and resulted in an augmented invasion of cancer cells. However, in cell lines with a vesicular AHNAK2 staining, such changes were not observed. Our study posits AHNAK2 as a valuable diagnostic biomarker in PDAC, now demanding prospective validation. Determination of mechanisms regulating AHNAK2 subcellular localisation may help explain its biological role.

## Introduction

Pancreatic ductal adenocarcinoma (PDAC) continues to be a diagnostic and therapeutic challenge, with little improvement to mortality in decades^1,2^. The diagnosis of PDAC is associated with poor prognosis due to several factors^3^. Low incidence (~ 12 per 100,000)^4^, non-specific and late symptoms, aggressive and resistant-to-treatment tumour biology with early-onset distant metastasis and finally, lack of sensitive and/or specific diagnostic biomarkers or imaging for early disease contribute to high mortality associated with this disease. From first cellular mutations to overt metastasis, PDAC develops over decades; yet most patients die an average of two years following the acquisition of metastatic capacity^5^, which usually arises around the time of diagnosis. Three main pre-malignant lesions develop into PDAC; pancreatic intraepithelial neoplasia (PanIN), intraductal papillary mucinous neoplasm (IPMN) and mucinous cystic neoplasm (MCN)^6,7^. Surgical resection can cure patients, particularly if IPMN and MCN are diagnosed at pre-invasive stage^8^. Carbohydrate antigen 19-9 (CA19-9), and carcinoembryonic antigen (CEA) are the only biomarkers for PDAC used in clinical practice. Their low sensitivity and specificity (78.2% and 82.8% for CA19-9) precludes use as a screening or diagnostic biomarker, such as for diagnosis of pre-malignant lesions, but they do have a role in measuring disease response and surveillance after treatment^4^.

AHNAK Nucleoprotein-2 (*AHNAK2*, neuroblast differentiation associated protein), was a component of gene panels developed for early diagnosis of pancreatic cancer^9^. High *AHNAK2* mRNA expression correlates with poor overall survival in multiple cancers, including clear cell renal carcinoma^10^, lung adenocarcinoma^11^, gastric cancer^12^, bladder cancer^13^ and uveal melanoma^14^. *AHNAK2*, initially designated *C14orf78*, was discovered in 2004 while exploring the function of its sister protein AHNAK nucleoprotein (AHNAK) in an *AHNAK* knock-out mouse model^15^. AHNAK2, is a large protein (616 kDa, 5795 amino acids) transcribed from chromosome 14q32 with a 15 kb open reading frame (ORF)^15^. Two further isoforms have been identified, isoforms 2 and 3, with a mass of 85 kDa and 605 kDa respectively^16,17^. The functional role of AHNAK2 remains unclear, with early evidence pointing towards roles in HIF-1α mediated epithelial-mesenchymal transition in hypoxic conditions^18^ and PI3K/AKT/mTOR mediated increase in tumour proliferation, migration and survival^10,14^. Here we explored the role of AHNAK2 in pancreatic cancer, as well as determining its potential utility as a biomarker for PDAC.

## Methods

### In silico analysis

PDAC tissue profiles from 177 patients taken from The Cancer Genome Atlas (TCGA) were analysed using cBioPortal.org^19^ and KMplot^20^ using Gene Set Enrichment Analysis (GSEA)^21^ of high (n = 267) versus low (n = 266) AHNAK2 mRNA expression data from TCGA. Positively and negatively correlated genes were grouped into Gene Set Ontologies. Only statistically significant Ontologies are presented.

### Enzyme linked immunosorbent assay (ELISA)

Blinded plasma samples, from 30 PDAC patients alongside 30 demographic-matched controls with no malignant or pancreatic disease, obtained from the Barts Pancreas Tissue Bank (BPTB; bartspancreastissuebank.org.uk; 2019/11/QM/HK/P/Blood and 2021/04/QM/HK/E/Blood&Tissue) were used to measure AHNAK2 concentration by ELISA following manufacturer’s instructions (bx501322, Abbexa, UK).

### Immunohistochemistry

4 µm sections of human PDAC tissue from 14 patients obtained from the BPTB (2021/04/QM/HK/E/Blood&Tissue) and human breast skin (positive control) were dewaxed, subject to antigen retrieval (100 mM tri-sodium citrate buffer, pH6) in a microwave (DeLonghi, 1200–1270 W, 20 min), quenched of endogenous peroxidase (methanol/3% hydrogen peroxide (H_2_O_2_), 15 min), washed (0.025% Triton X100 in Tris-buffered saline (TBS)), blocked (goat serum in 5% bovine serum albumin (BSA) (1:25), 1 h at RT), incubated with anti-AHNAK2 antibody (Supplementary Tables 1, 2) or an IgG isotype or TBS control, washed, incubated with the secondary antibody, washed and signal amplified with VECTASTAIN® peroxidase anti-rabbit IgG avidin–biotin complex (ABC) kit (PK-6105), before developing colour with 3,3′-Diaminobenzidine (DAB) substrate (PK-6105, 2 min), and finally counterstaining with haematoxylin. Quantification of IHC was performed using QuPath^22^.

### Cell culture

Cancer (Supplementary Table 3) and pancreatic stellate (PS1^23^) cells were cultured in separate 6-, 12- and 24-well culture plates, depending on experimental conditions, on and without glass coverslips (VWR) using their respective media, having being short tandem repeat (STR) profiled and tested negative for mycoplasma.

### Immunofluorescence

Cells were fixed (0.1% formalin (Cellpath PlC), 10 min), blocked (PBS containing 5% Goat Serum and 0.3% TritonX100) and incubated with anti-AHNAK2, anti-E-cadherin, anti-Ezrin, anti-Cortactin or anti-Vimentin antibodies (Supplementary Table 2), alongside relevant isotype negative control. Alexa-Fluor goat anti-rabbit 488 and Alexa-Fluor goat anti-mouse 546 were used as secondary antibodies. Alexa Fluor 647 Phalloidin was used to stain for filamentous actin. Coverslips were mounted with anti-fade along with DAPI (P36931, ThermoFisher UK), and imaged with an LSM 710 confocal microscope (Leica Biosystems). Quantification of expression was performed using CellProfiler^24^ using a universal pipeline that separates each channel through ‘Image processing’ then separates each cell with ‘Object Processing’ using the nucleus for identification. ‘MeasureImageIntensity’ was used for calculating the Arbitrary Intensity Units (AU) and ‘MeasureColocalisation’ for the overlap coefficient using object-based (per cell) co-localisation.

### Real time quantitative polymerase chain reaction (RT-qPCR)

RNA was extracted using the Quick-RNA miniprep kit (Zymo research, CA, USA), quantified (nanodrop ND-1000 spectrophotometer), cDNA synthesised (1 µg of RNA, SuperScript III Reverse Transcriptase (Invitrogen, CA, USA) according to manufacturer’s instructions. RT-qPCR was performed using QuantiTect pre-designed primers (Qiagen, CA, USA), SensiFAST SYBR® HI-ROX kit (BIO-92005, Bioline) and a StepOnePlus Real Time PCRsystem (ThermoFisher Scientific) according to manufacturer’s instructions. Results were normalised to GAPDH in normoxia and RP2^25^ in hypoxic conditions and calculated using the ΔΔCT method^26^ for normalised relative expression. PDK primers were used to confirm a hypoxic phenotype in cell lines^27^.

### Hypoxia

In hypoxia experiments, identical plates of cells were incubated for 30 min in a normoxic incubator to allow cells to adhere, before being left in normoxia or inserted into a hypoxic incubator (Baker Invivo2 400 workstation) at 37 °C, 1% O_2_ and 5% CO_2_ for 24 h before extracting RNA or fixation of cover slips. RNA extraction occurred in the hypoxic incubator at the end of the experiment. In experiments that would take multiple days, hypoxic cells would be in a hypoxic incubator for the last 24 h.

### siRNA transfection

Cells were transfected with 10 nM *AHNAK2* siRNA (231784, Ambion, USA) (NCBI Reference NM_138420.2) in INTERFERin® (101000016, Polyplus, France) according to manufacturer’s protocol (10 min, RT), followed by incubation in serum containing media for 24 h (day 2) and 48 h (day 3) before RNA extraction, seeding on coverslips, or use in spheroid and substrate experiments (incubated for 24 h).

### Cellular substrate experiments

Coverslips and wells were coated with the appropriate substrate (50 μl low concentration Collagen (354236, Corning®, USA), 50 μl Fibronectin (F0895, Sigma, USA) or 50 μl Matrigel (356234, Corning®, USA)). Following incubation in both normoxic and hypoxic conditions (incubated for 30 min in a normoxic incubator before being inserted into a hypoxic incubator (Baker Invivo2 400 workstation) at 37 °C, 1% O_2_ and 5% CO_2_) for 4 h, then fixed with 0.1% formalin (Cellpath PLC) or lysed for RNA extraction.

### Spheroid model

1000 cancer cells were co-cultured with 2,000 pancreatic stellate cells (PS1) in a 1:2 ratio in 2.5% (v/v) methylcellulose (M0512, Sigma, USA) solution in hanging droplets for 24 h to allow formation of cellular spheroids as described previously^28^. A 96-well plate was pre-coated (30 min at 37 °C) with extra-cellular matrix (ECM) gel mixture consisting of 10.5 volumes high concentration Collagen (354249, Corning®, USA) with a 2 mg/ml final concentration, 7 volumes Matrigel (356234, Corning®, USA), 1 volume 25 mM HEPES (H7006, Sigma, USA) and 21.5 volumes relevant cell culture medium plus sodium hydroxide to neutralise the pH. Six spheres were collected using a cut pipette tip, washed in medium then suspended in ECM gel mixture. 200 μl of appropriate medium was added to the top of the gels and the spheroids were incubated for three days and imaged for invasion. Gels were imaged using an Axiovert 135 (Carl Zeiss MicroImaging LLC) camera and the percentage invasive area quantified using ImageJ (National Institutes of Health) with the equation: % invasive area = ((total area − central area)/central area) × 100.

### Proliferation assay

2500 cancer cells were seeded in the central 64 wells of a 96-well plate in triplicates, 200 µl PBS was added to the outside wells to prevent dehydration within the plate during incubation impacting on results. 90 μl relevant medium was added per well together with 20 μl MTT solution (Invitrogen™, Thermo Fisher). Cells were incubated concurrently in a normoxic or hypoxic incubator (2 h), and absorbance (550 nm) was read using a 96-well microplate reader (Infinite® F50, Magellan software).

### Statistical analysis

Analyses were performed using GraphPad Prism (Version 8.0.0) and R (version 3.5.1) using package ROCR^29^ for the Area Under the Receiver Operating Characteristics (AUROC) with appropriate statistical tests as described.

## Results

### AHNAK2 expression is increased in PDAC tissue and is associated with hypoxia

**Fig. 1:**
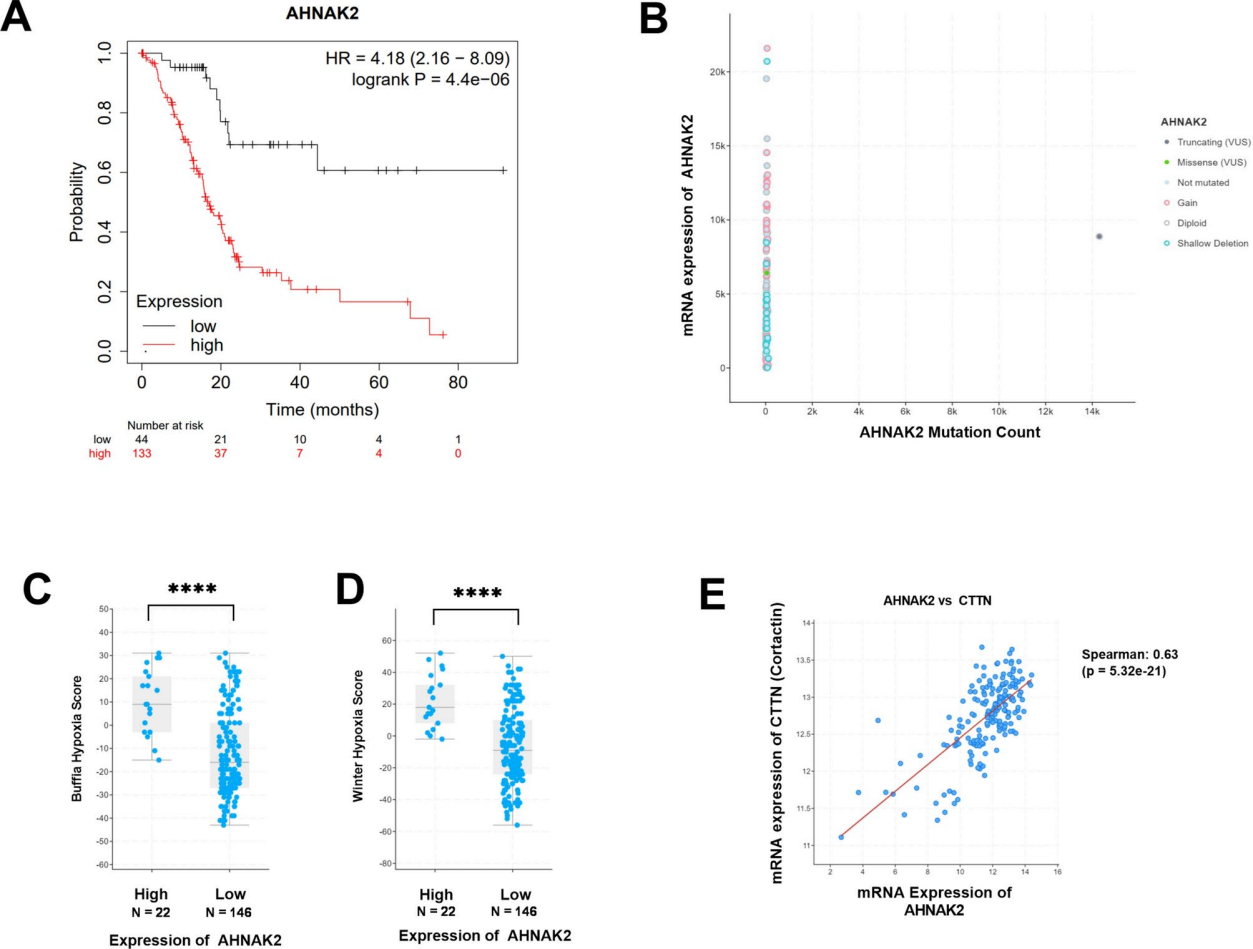
In silico AHNAK2 expression in human PDAC samples. (**A**) A Kaplan–Meier survival curve of 177 patients with PDAC separated according to *AHNAK2* expression. (**B**) Mutation and mRNA expression of AHNAK2. (**C**, **D**) Hypoxia scores and *AHNAK2* mRNA expression. *AHNAK2* expression separated with Z-score threshold*.* (**E**) MSigDB gene set ontology correlation analysis of *AHNAK2* and *Cortactin* gene expression in tissues of patients with PDAC. Log-rank test (**A**), Mann–Whitney *U* test (**C**, **D**), Pearson test for correlation (**E**). ****, p < 0.0001.

In silico analysis of The Cancer Genome Atlas (TCGA) dataset (n = 177 PDAC patient tissues)^30^, using cBioPortal^19^ showed that high *AHNAK2* mRNA expression was associated with poor prognosis after surgical resection of pancreatic cancer (Fig. 1A). Whilst there was one patient with mutations in *AHNAK2* (n = 174), there were significant numbers of patients with increased expression (Fig. 1B). High *AHNAK2* expression was associated with higher Buffa and Winter hypoxia scores^31,32^ (Fig. 1C,D), suggesting *AHNAK2* may have either a role in the development of a hypoxic milieu or *AHNAK2* expression is increased due to hypoxia. Gene Set Enrichment Analysis (GSEA)^21^ indicated cellular adhesion, cellular junctions and embryological development as the most likely biological functions related to high *AHNAK2* expression (Supplementary Table 4). *Cortactin* expression was found to be positively correlated to *AHNAK2* expression (Fig. 1E). Genes negatively correlated with *AHNAK2* were associated with cytoplasmic vesicle formation and cellular metabolic processes (Supplementary Table 5).

*AHNAK2* showed high variability in mRNA expression as well as protein level and distribution in a range of pancreatic cancer cell lines (Supplementary Figs. 1–3). We observed vesicular cytoplasmic distribution of AHNAK2 in cell lines having low mRNA expression (e.g., COLO357) while those cell lines demonstrating higher mRNA and protein expression showed high AHNAK2 co-localisation with Cortactin and Ezrin at cellular protrusions (e.g., MIAPaCa-2, Capan-2).

### AHNAK2 and cortactin co-localise at cellular protrusions in response to extra-cellular matrix

Since the expression and co-localisation of Ezrin and Cortactin may change in response to underlying extra-cellular matrix (ECM) proteins and result in development of cellular protrusions^33,34^, we investigated changes in *AHNAK2* mRNA and protein expression and localisation in response to distinct ECM proteins commonly found in PDAC stroma^35,36^. Matrigel, consisting of Collagen IV, Entactin, Perlecan and Laminin^37^ was used as control representing basement membrane, whilst Fibronectin and Collagen were used to represent ECM proteins in pathological tissues.

**Fig. 2:**
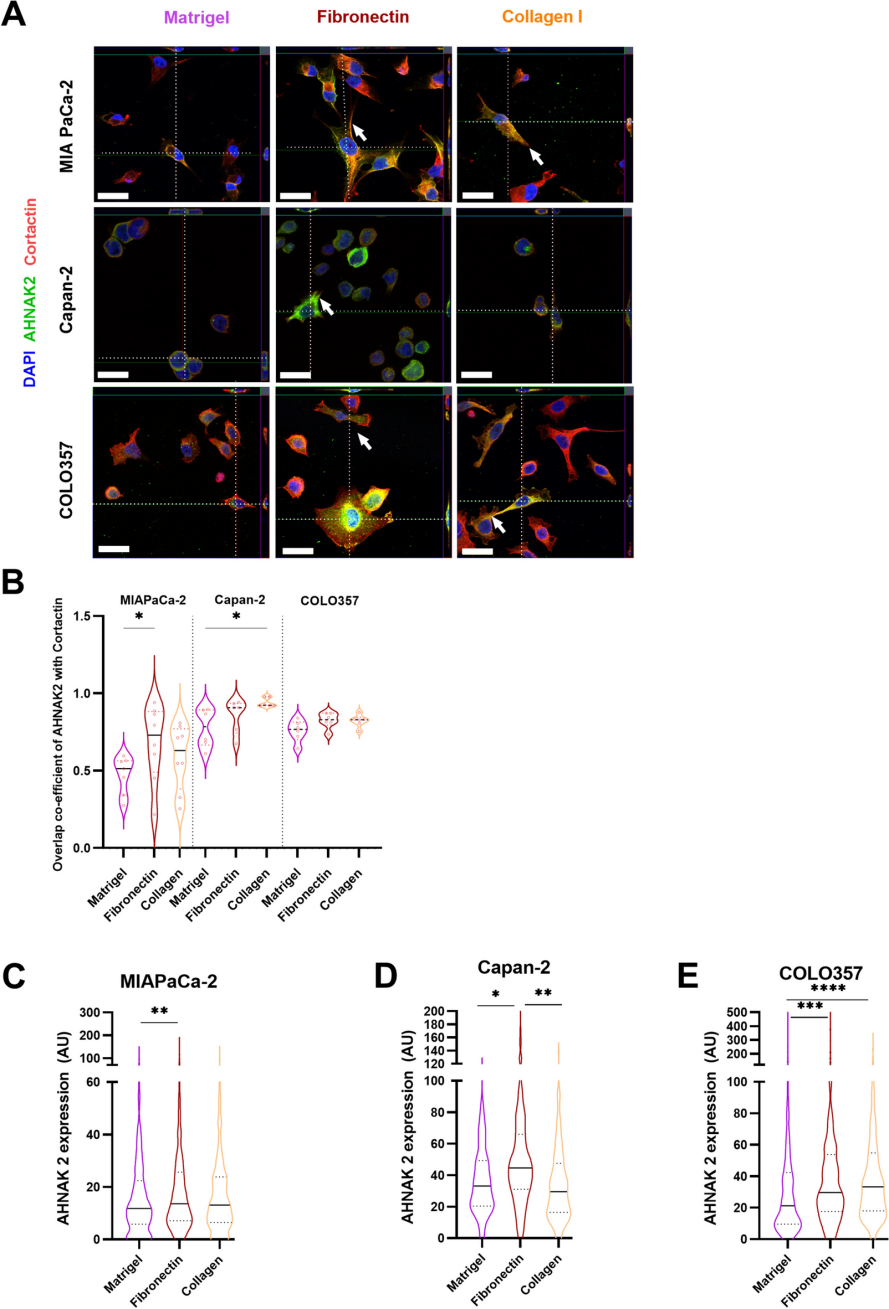
ECM proteins modify AHNAK2 expression and its co-localisation with Cortactin. (**A**) Z-stack images of cancer cell lines on three different substrates with immune-staining as shown. Arrows point to cellular processes (pseudopodia, filipodia, lamellipodia). (**B**) CellProfiler co-localisation tool assessment of the ‘overlap co-efficient’ (modification of Pearson’s correlation coefficient) of AHNAK2 with Cortactin. Each point represents a summation of multiple cells from a single image taken (n = 3 biological repeats). (**C**–**E**) Quantification of total AHNAK2 protein expression per cell from three biological repeats (minimum 1000 cells). Kruskal–Wallis test with post-hoc Dunn test. *p < 0.05; **p < 0.01; ***p < 0.001; ****p < 0.0001. Scale bar = 10 μm.

Whilst *AHNAK2* mRNA expression did not change (Supplementary Fig. 10, its protein expression and localisation in cellular processes altered significantly (Fig. 2A). For example, in the MIA PaCa-2 cell line, AHNAK2 protein expression was significantly higher in response to Fibronectin in comparison to Matrigel, with greater co-localisation with Cortactin (Fig. 2B,C), with similar changes seen for Capan-2 cells (Fig. 2B,D). In contrast low-AHNAK2 expressing COLO357 cells, showed increased expression of AHNAK2 when cultured on either Collagen I or Fibronectin but no change in co-localisation with Cortactin (Fig. 2B,E). Co-localisation with Cortactin was seen mainly at the cellular protrusions (filopodia, pseudopodia, lamellopodia) which appeared when cells were grown on Fibronectin and Collagen I, with fewer cells exhibiting a vesicular pattern of expression of AHNAK2 in these conditions (Fig. 2A).

### AHNAK2 and Cortactin co-localise and form cellular protrusions in hypoxia

**Fig. 3:**
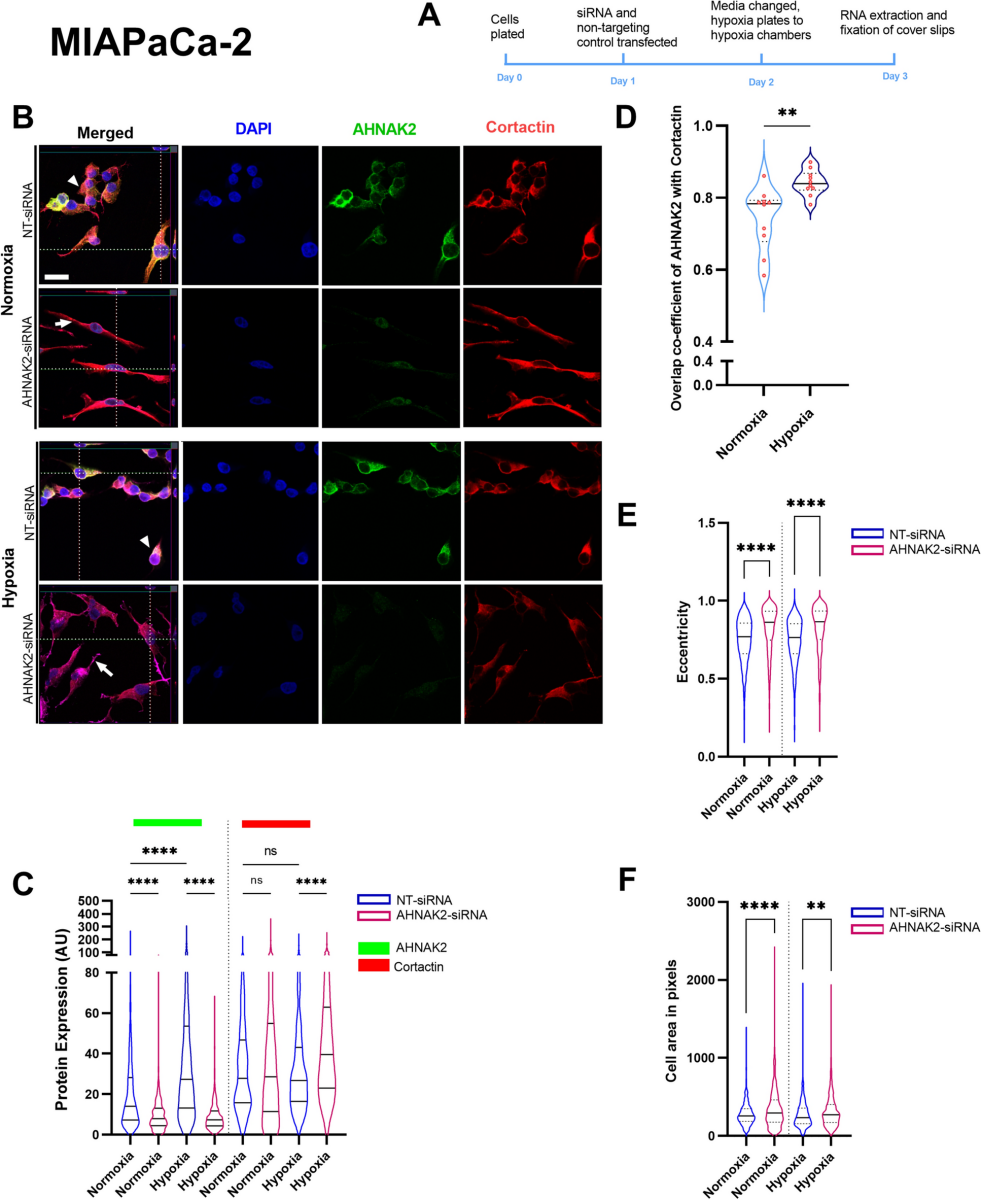
Hypoxia increases co-localisation of AHNAK2 and Cortactin in MIA PaCa-2 cells with change in cell shape. (**A**) Timeline of the experiment. (**B**) Z-stack images of MIAPaCa-2 cell line after AHNAK2-siRNA along with non-targeting (NT) control siRNA in normoxic and hypoxic conditions to show change in the cellular phenotype such as thinner cells apico-basally (Z-stack), elongated cells after AHNAK2-siRNA. Arrows suggest filipodia and, white arrow heads point at the pseudopodia. (**C**) AHNAK2 and Cortactin protein expression changes as quantified by CellProfiler. (**D**) Co-localisation of AHNAK2 and Cortactin in normoxia and hypoxia. Quantification of cell shape or eccentricity (**E**) and area (**F**) with *AHNAK2*-siRNA and normoxic and hypoxic conditions. Each experiment had a minimum of three biological repeats and each violin plot contains a minimum of 1000 quantified cells. Kruskal–Wallis test with post-hoc Dunn test (**C**) and Mann–Whitney U test (**D**–**F**). ns, not significant; **p < 0.01; ****p < 0.0001. Scale bar = 10 μm.

MIA PaCa-2 cells showed increased expression of Cortactin and Ezrin, with a significant increase in AHNAK2 expression in hypoxic conditions, as compared to normoxia (Fig. 3, Supplementary Fig. 4). In hypoxic conditions there was increased co-localisation of AHNAK2 and Cortactin (Fig. 3D). *AHNAK2* knockdown resulted in increased Cortactin expression in hypoxic conditions (Fig. 3C). *AHNAK2* siRNA knockdown led to a change in cellular phenotype with spindle-shaped cells and long cellular protrusions (Fig. 3B), as measured by higher eccentricity, larger cell volume and longer axis (Fig. 3E,F).

**Fig. 4:**
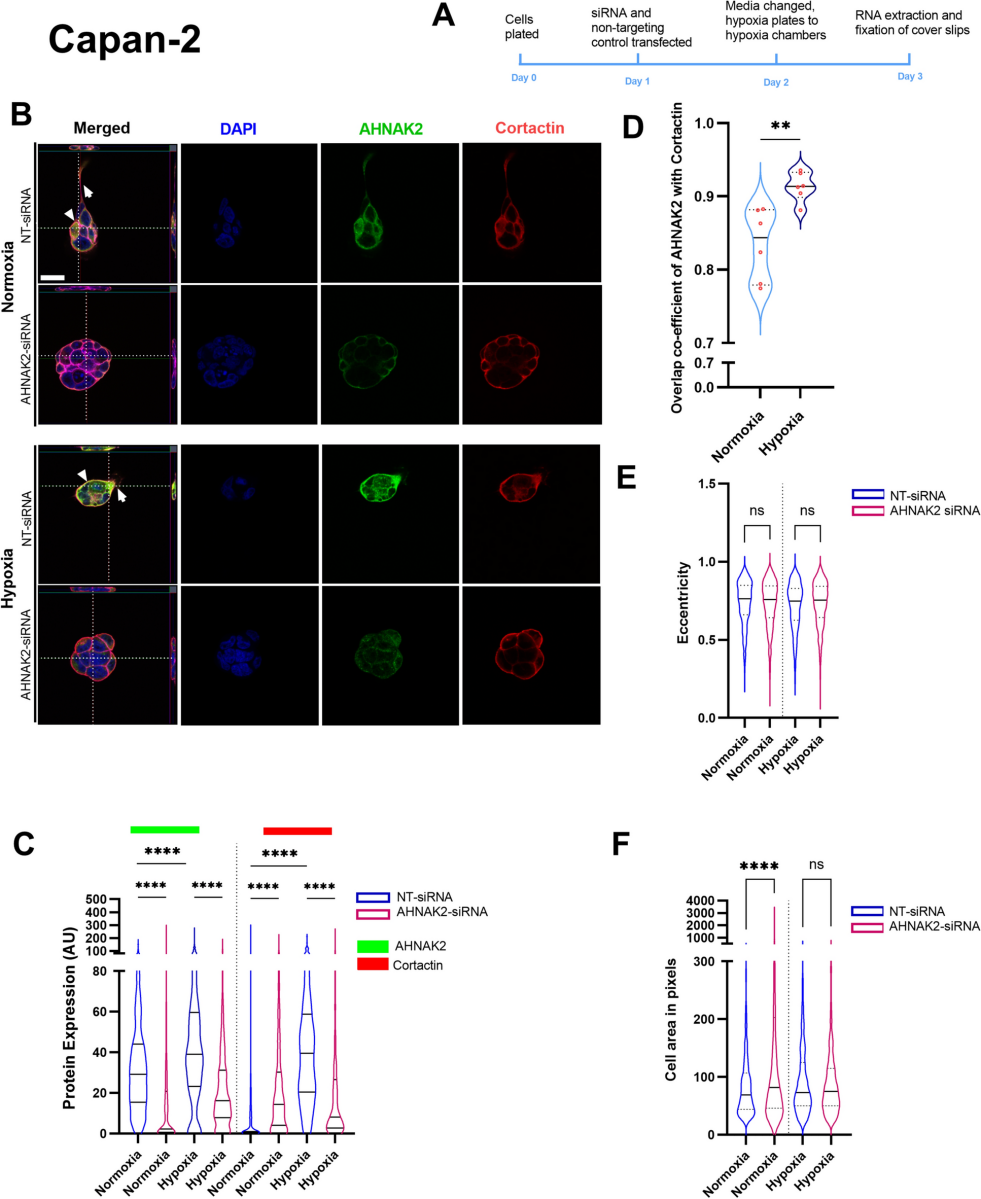
Hypoxia increases co-localisation of AHNAK2 and Cortactin in Capan-2 cells, with no change in cell shape. (**A**) Timeline schema of the experiment. (**B**) Z-stack images of Capan-2 cell line after *AHNAK2*-siRNA along with non-targeting (NT) control siRNA in normoxic and hypoxic conditions to show change in the cellular phenotype. Arrows suggest filipodia and, white arrow heads point at the pseudopodia. (**C**) AHNAK2 and Cortactin protein expression changes as quantified by CellProfiler. (**D**) Co-localisation of AHNAK2 and Cortactin in normoxia and hypoxia. Quantification of cell shape or eccentricity (**E**) and area (**F**) with AHNAK2-siRNA and normoxic and hypoxic conditions. Each experiment had a minimum of three biological repeats and each violin plot contains a minimum of 1000 quantified cells. Kruskal–Wallis test with post-hoc Dunn test (**C**) and Mann–Whitney *U* test (**D**–**F**). ns, not significant; **p < 0.01; ****p < 0.0001. Scale bar = 10 μm.

**Fig. 5:**
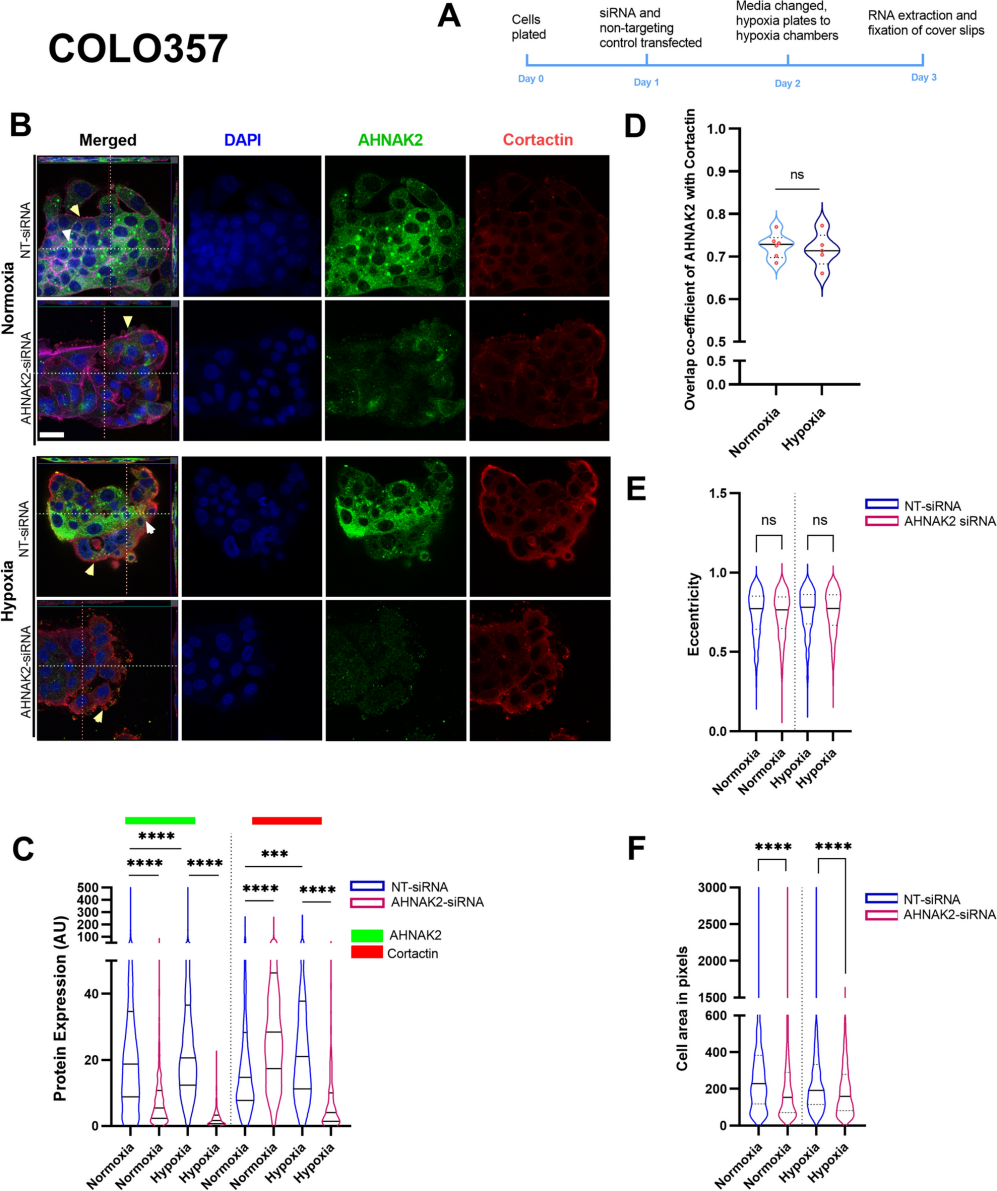
Effect of hypoxia in cellular localisation of AHNAK2 and Cortactin in COLO357 cells. (**A**) Timeline schema of the experiment. (**B**) Z-stack images of COLO357 cell line after *AHNAK2*-siRNA along with non-targeting (NT) control siRNA in normoxic and hypoxic conditions to show change in the cellular phenotype. Yellow arrows point to changes in continuity of the cellular membrane and, white arrow heads point at the pseudopodia. (**C**) AHNAK2 and Cortactin protein expression changes as quantified by CellProfiler. (**D**) Co-localisation of AHNAK2 and Cortactin in normoxia and hypoxia. Quantification of cell shape or eccentricity (**E**) and area (**F**) with *AHNAK2*-siRNA and normoxic and hypoxic conditions. Each experiment had a minimum of three biological repeats and each violin plot contains a minimum of 1000 quantified cells. Kruskal–Wallis test with post-hoc Dunn test (**C**) and Mann–Whitney *U* test (**D**–**F**). ns, not significant; ***p < 0.001; ****p < 0.0001. Scale bar = 10 μm.

Capan-2 cells, growing as colonies with few cellular protrusions in normoxic conditions, exhibited numerous cellular protrusions in hypoxic conditions, with strong co-localisation of AHNAK2 and Cortactin as well as Ezrin (Fig. 4, Supplementary Fig. 5). AHNAK2 and Cortactin expression increased in hypoxia, with a decrease in Ezrin expression (Supplementary Fig. 5), resulting greater co-localisation of AHNAK2 with Cortactin (Fig. 4B–D). There was no change in cell eccentricity in either condition after *AHNAK2* siRNA-mediated knockdown (Fig. 4E). The increase in cell size in normoxic conditions after *AHNAK2* knockdown was not observed in hypoxic conditions (Fig. 5F), perhaps due to a surprising decrease in *AHNAK2* mRNA in hypoxia (Supplementary Fig. 5D) and Cortactin protein expression (Fig. 4C), in contrast to that observed for MIA PaCA-2 cells.

Whilst AHNAK2 expression increased significantly, along with Cortactin and Ezrin expression, in COLO357 cells incubated in hypoxic conditions (Fig. 5, Supplementary Fig. 6), there was no change to co-localisation of AHNAK2 with Cortactin, with AHNAK2 showing primarily cytoplasmic distribution (Fig. 5B–D), and these cells retaining epithelial colony formation, in contrast to Capan2 cells. As such, cell morphology (elongation) did not change (Fig. 5E), but cells became smaller (Fig. 5F) after *AHNAK2* knockdown in either hypoxic or normoxic conditions, with an interrupted Cortactin deposition at colony border in hypoxic conditions suggesting a change in cell membrane/underlying cytoskeleton, especially after *AHNAK2* knockdown (Fig. 5B). Experimental findings are summarised in the Supplementary data (Supplementary Tables 6–8). Taken together, our findings implicate distinct mechanisms regulating Cortactin, Ezrin and AHNAK2 expression in different PDAC cell lines in normoxic and hypoxic conditions, with a critical role for AHNAK2 alongside Cortactin in cell morphology. This led us to investigate the impact of AHNAK2 on cell motility.

### AHNAK2 increases invasion in Capan-2 PDAC cells and has minimal effect on proliferation

**Fig. 6:**
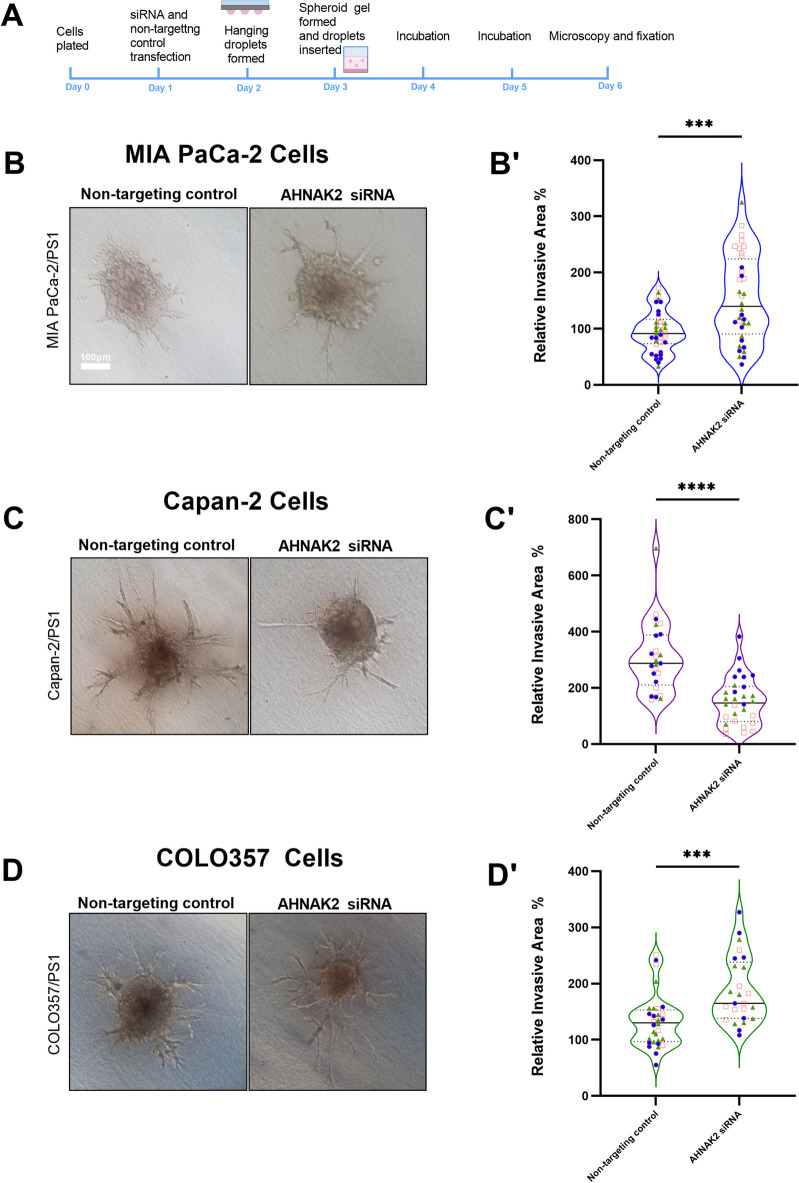
AHNAK2 knockdown reduces invasion in Capan-2. (**A**) Schema for spheroid co-culture experiment. Examples of MIAPaCa-2/PS1 (**B**), Capan-2/PS1 (**C**) and COLO357/PS1 (**D**) spheroids transfected with non-targeting siRNA and *AHNAK2* siRNA after 48h of incubation. Quantification of relative invasive area of co-cultured MIAPACA-2 (**B’**) Capan-2 (**C’**), COLO357 (**D’**). Each point represents one spheroid with n = 3 biological repeats represented with different symbols. Mann–Whitney *U* test (**B’**–**D’**) ***p < 0.001; ****p < 0.0001. Scale bar = 100 µm.

Proliferation rates after hypoxia were unchanged in all three cell lines, despite effective silencing (Supplementary Fig. 8). To assess the effect of loss of AHNAK2 upon cancer cell motility, we used a hanging drop spheroid invasion assay developed in our laboratory, allowing assessment of 3D physiomimetic invasion^38^ (Fig. 6A). All three cancer cell lines with *AHNAK2* knockdown or non-targeting siRNA controls, were co-cultured individually with pancreatic stellate cells (PS1). Invasion of Capan-2 cells was reduced significantly following *AHNAK2* knockdown (Fig. 6B–G), but reverse was seen for MIA PaCa2 and COLO 357 which have different basal expression and co-localisation.

### AHNAK2 is significantly higher in PDAC patient blood and tissue compared to normal controls

**Fig. 7:**
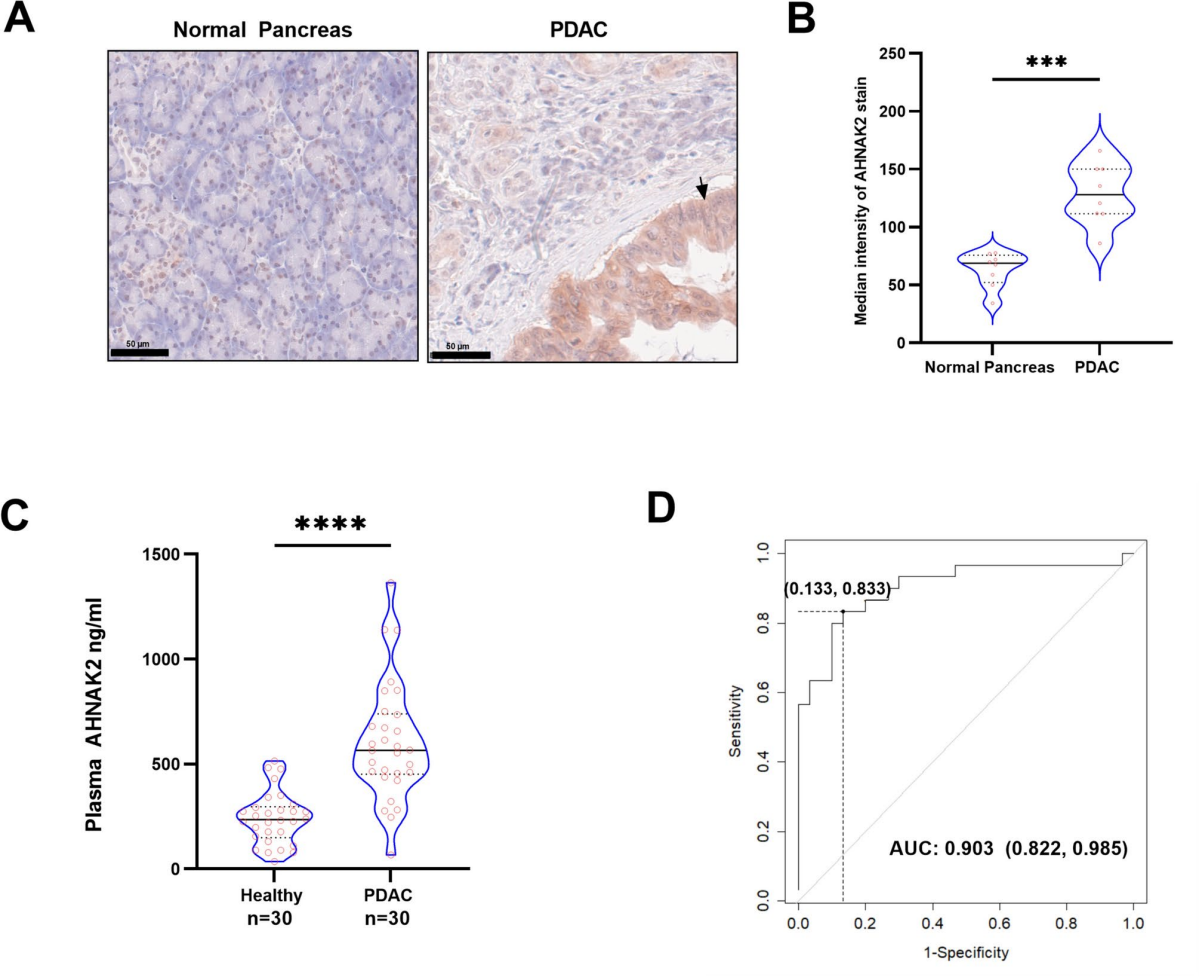
AHNAK2 expression is significantly increased in tissue and serum of patients with PDAC. (**A**) Tissue expression of AHNAK2 in normal pancreas and PDAC. Arrows point towards pancreatic cancer cells. (**B**) IHC stain quantification with QuPath of TMA cores of PDAC and normal pancreas tissue (N = 8). (**C**) Plasma concentration of AHNAK2 in 30 PDAC patients and 30 healthy controls. (**D**) A ROC curve with plasma AHNAK2 concentration of 421.47 ng/ml with a specificity and sensitivity of 83.33% and 86.67% respectively and an AUC of 0.903 [0.822, 0.985]. Mann–Whitney *U* Test (**B**, **C**), bootstrapped standard error was used for significance of the AUROC (**D**), ***p < 0.001; ****p < 0.0001, Scale bar = 50 µm.

AHNAK2 expression in PDAC tissue has been previously described as mainly cytoplasmic and membranous in epithelial cancer cells^39^. Immunohistochemistry of 14 samples of resected human PDAC showed protein expression in cancer cells (Fig. 7), with adjacent normal pancreas demonstrating no expression of AHNAK2 (Fig. 7). Higher AHNAK2 protein expression was also observed in plasma samples of patients with PDAC (n = 30) compared to age-and gender-matched controls (n = 30) (Fig. 7B). Using a threshold of 421.47 ng/ml, AHNAK2 expression could potentially diagnose PDAC with a specificity and sensitivity of 83.33% and 86.67% respectively (Fig. 7C). However, in this small cohort, there was no correlation between AHNAK2 plasma levels and tissue expression levels (Supplementary Fig. 9).

## Discussion

### AHNAK2 is an oncogenic protein in PDAC

We chose *AHNAK2* as a potential oncogene and diagnostic biomarker based on its inclusion in multiple gene-panels used to discriminate between PDAC patients and healthy controls through laser-dissected pancreatic tissue^40^. We confirmed this increased expression in PDAC tissue in publicly available datasets of patients with PDAC (TCGA), but no *AHNAK2* mutations were reported in the data. Furthermore, we confirmed high protein expression in tissues and plasma of patients with PDAC as compared to healthy counterparts. This implies other factors playing a role for this enhanced expression, such as association with hypoxia, leading to it being a poor prognostic factor. AHNAK2, normally described as a nucleoprotein, was found in the cytoplasm in almost all pancreatic cancer cell lines. In ccRCC, HIF-1α mediated AHNAK2 upregulation was demonstrated in hypoxia, inducing epithelial-mesenchymal transition (EMT)^10^. Similar results found AHNAK2 as a promoter of invasion and EMT in ung adenocarcinoma cell lines mediated through the TGF-β/Smad3 Pathway^41^. Since, AHNAK2 is a large protein of 616 kDa and is similar in structure to AHNAK, it likely also forms part of large multi-protein complexes and scaffolding networks. In fact, GSEA of the TCGA cohort revealed multiple genes positively correlated with AHNAK2 overexpression, including Ezrin and Cortactin. Cortactin and Ezrin are an essential part of the EMT machinery leading to migration and invasion by participation in formation of cellular processes^34,42,43^.

Cortactin, along with Ezrin, two distinct cytoskeletal organising proteins, are critical in the formation of cellular processes in PDAC, which may aid in invasion and metastasis of cancer cells^34^. Since AHNAK2 protein structure is highly suggestive of a cytoskeletal protein^40^, it may interact with Cortactin and be involved in the formation of cellular protrusions. Taken together, these in silico analyses indicate that high *AHNAK2* expression may be associated with poor survival due to its role in the invasive capability of cancer cells, which is potentiated by hypoxia.

The heterogenous AHNAK2 sub-cellular localisation and how its association with Ezrin and Cortactin changed in response to extra-cellular matrix and hypoxia was, perhaps, the key factor in at least two distinct phenotypes: vesicular or diffuse cytoplasmic localisation. In cells with diffuse cytoplasmic AHNAK2 there was co-localisation with Cortactin in cellular protrusions. Vesicular localisation of AHNAK2, not associated with cellular protrusions, looked similar to phagosomes identified with microtubule-associated protein 1 light chain 3 (LC3) and Lysosomal-associated membrane protein 1 (LAMP-1) in other studies^44,45^. The role of autophagy in tumour growth in PDAC has been reported^46^ and therefore AHNAK2 may partake in that mechanism in a subset of cancer cell lines such as COLO357. However, we limited our study to the interaction between AHNAK2 and Cortactin in normoxia and hypoxia, focusing on invasion and motility. We included COLO357 as a cell line with vesicular expression in our experiments as a control since this phenotype may potentially behave differently.

In PDAC, hypoxia promotes EMT and invasion of cancer cells^47^ through a range of molecular mechanisms. We could demonstrate, that in addition to other molecular pathways leading to phenotypic and functional cellular change, AHNAK2 protein expression was increased in hypoxia with an increase co-localisation in cellular process along with Cortactin, contributing to the mesenchymal phenotype, at least in some of cancer cell lines where there was cytoplasmic and not vesicular distribution of AHNAK2 during normoxia. Although the exact mechanisms are not elucidated in this research, other groups have shown *AHNAK2* knockdown impairs hypoxia-induced EMT and stem cell-like properties^10^. This is an important avenue for further research.

Whilst in PDAC cell lines there was no change in proliferation, in uveal melanoma (UM) and Thyroid Cancer (TC) AHNAK2 upregulates the PI3K/AKT pathway, known to regulate mTOR and thereby controlling proliferation, growth and survival^10,14,48^. *AHNAK2* knockdown supressed proliferation, migration and invasion in UM and TC. Lastly, presence of extra-cellular matrix proteins, abundantly seen in the tumour micro-environment of PDAC, could also enhance cellular process co-localisation of AHNAK2 with Cortactin. In the Capan-2 cell line, *AHNAK2* knockdown significantly reduced invasion, which is interesting as only Capan-2 cells, and not COLO357 or MIA PaCa2 cells show a more epithelial phenotype with *AHNAK2*-siRNA transfection. As Capan-2 originates lymph node metastasis from a well-differentiated cancer^49^, this could represent variable AHNAK2 expression based on tumour grade and may indicate suitability as a therapeutic target in specific tumour stage and mutational burden.

### AHNAK2 as a clinical biomarker

CA19-9 is currently the only clinically validated biomarker used in PDAC for disease response monitoring and surveillance. As a diagnostic biomarker, it achieves a median sensitivity and specificity of 79% and 82%^50^, and thus is not used for diagnosis. Our pilot study with 30 healthy controls and 30 PDAC patients found that AHNAK2 expression in plasma achieved a sensitivity and specificity of 86.67% and 83.33% with an AUC of 0.903. This outperformance over CA19-9 needs to be validated in a large sample size, controlling for other variables such as non-secretion of CA19-9 in Lewis genotype negative^50,51^, as well as co-morbidities, lifestyle factors, ethnicity and other unknown confounders. Our limited analysis has shown that neither age, tumoral AHNAK2 expression or tumour stage influence AHNAK2 levels in plasma. Furthermore, low AHNAK2 plasma level reflected poorer survival outcome, compared to high tumoral AHNAK2 mRNA leading to poor prognosis. Although AHNAK was found to be secreted by mammary carcinoma cell lines^52^ we have not explored in vitro AHNAK2 secretion for the cell lines studied. Thus, the overall contribution of tumoral AHNAK2 to its plasma levels will need more in-depth study. This initial report suggests the AHNAK2 may act as oncoprotein and may function as a potential diagnostic and prognostic biomarker.

## Supplementary Information

Supplementary Information.

## Data Availability

Data are available upon reasonable request.

## References

[1] Rahib, L. et al. Projecting cancer incidence and deaths to 2030: The unexpected burden of thyroid, liver, and pancreas cancers in the United States. *Cancer Res.* **74**(11), 2913–2921 (2014). 24840647 10.1158/0008-5472.CAN-14-0155

[2] Kleeff, J. et al. Pancreatic cancer. *Nat. Rev. Dis. Primer.* **2**(1), 1–22 (2016). 10.1038/nrdp.2016.22 27158978

[3] Cancer statistics, 2019. *CA Cancer J. Clin*. 10.3322/caac.21551.

[4] Singhi, A. D., Koay, E. J., Chari, S. T. & Maitra, A. Early detection of pancreatic cancer: Opportunities and challenges. *Gastroenterology.* **156**(7), 2024–2040 (2019). 30721664 10.1053/j.gastro.2019.01.259 PMC6486851

[5] Yachida, S. et al. Distant metastasis occurs late during the genetic evolution of pancreatic cancer. *Nature.* **467**(7319), 1114–1117 (2010). 20981102 10.1038/nature09515 PMC3148940

[6] Tanaka, M. Thirty years of experience with intraductal papillary mucinous neoplasm of the pancreas: From discovery to international consensus. *Digestion.* **90**(4), 265–272 (2014). 25591885 10.1159/000370111

[7] Komatsu, S. et al. Malignant potential in pancreatic neoplasm; New insights provided by circulating miR-223 in plasma. *Expert. Opin. Biol. Ther.* **15**(6), 773–785 (2015). 25819175 10.1517/14712598.2015.1029914

[8] Einama, T. et al. Curative resection of pancreatic ductal adenocarcinoma developing in the remnant pancreas 13 years after distal pancreatectomy for intraductal papillary mucinous neoplasms: A case report. *Mol. Clin. Oncol.* **8**(3), 417–420 (2018). 29456847 10.3892/mco.2018.1556 PMC5795772

[9] Klett, H. et al. Identification and validation of a diagnostic and prognostic multi-gene biomarker panel for pancreatic ductal adenocarcinoma. *Front. Genet.*10.3389/fgene.2018.00108 (2018). 29675033 10.3389/fgene.2018.00108 PMC5895731

[10] AHNAK2 is a novel prognostic marker and oncogenic protein for clear cell renal cell carcinoma. (2019). https://www.ncbi.nlm.nih.gov/pmc/articles/PMC5399579/. 10.7150/thno.18198 PMC5399579 28435451

[11] Zhang, S. et al. AHNAK2 is associated with poor prognosis and cell migration in lung adenocarcinoma. *BioMed. Res. Int.* **21**(2020), e8571932 (2020). 10.1155/2020/8571932 PMC7456490 32904605

[12] Ohmura, H. et al. Methylation of drug resistance-related genes in chemotherapy-sensitive Epstein-Barr virus-associated gastric cancer. *FEBS Open Biol.* **10**(1), 147–157 (2020). 10.1002/2211-5463.12765 PMC6943226 31736281

[13] Witzke, K. E. et al. Integrated Fourier transform infrared imaging and proteomics for identification of a candidate histochemical biomarker in bladder cancer. *Am. J. Pathol.* **189**(3), 619–631 (2019). 30770125 10.1016/j.ajpath.2018.11.018

[14] Li, M., Liu, Y., Meng, Y. & Zhu, Y. AHNAK nucleoprotein 2 performs a promoting role in the proliferation and migration of uveal melanoma cells. *Cancer Biother. Radiopharm.* **34**, 624–633 (2019). 10.1089/cbr.2019.2778 31621397

[15] Komuro, A. et al. The AHNAKs are a class of giant propeller-like proteins that associate with calcium channel proteins of cardiomyocytes and other cells. *Proc. Natl. Acad. Sci. USA.* **101**(12), 4053–4058 (2004). 15007166 10.1073/pnas.0308619101 PMC384694

[16] Gerhard, D. S. et al. The status, quality, and expansion of the NIH full-length cDNA project: The Mammalian Gene Collection (MGC). *Genome Res.* **14**(10B), 2121–2127 (2004). 15489334 10.1101/gr.2596504 PMC528928

[17] Ota, T. et al. Complete sequencing and characterization of 21,243 full-length human cDNAs. *Nat. Genet.* **36**(1), 40–45 (2004). 14702039 10.1038/ng1285

[18] Hapke, R. & Haake, S. M. Hypoxia-induced epithelial to mesenchymal transition in cancer. *Cancer Lett.* **1**(487), 10–20 (2020). 10.1016/j.canlet.2020.05.012 PMC7336507 32470488

[19] Cerami, E. et al. The cBio cancer genomics portal: An open platform for exploring multidimensional cancer genomics data. *Cancer Discov.* **2**(5), 401–404 (2012). 22588877 10.1158/2159-8290.CD-12-0095 PMC3956037

[20] Lánczky, A. & Győrffy, B. Web-based survival analysis tool tailored for medical research (KMplot): Development and Implementation. *J. Med. Internet Res.* **23**(7), e27633 (2021). 34309564 10.2196/27633 PMC8367126

[21] Subramanian, A. et al. Gene set enrichment analysis: A knowledge-based approach for interpreting genome-wide expression profiles. *Proc. Natl. Acad. Sci.* **102**(43), 15545–15550 (2005). 16199517 10.1073/pnas.0506580102 PMC1239896

[22] Bankhead, P. et al. QuPath: Open source software for digital pathology image analysis. *Sci. Rep.* **7**(1), 16878 (2017). 29203879 10.1038/s41598-017-17204-5 PMC5715110

[23] Wang, Q., Maher, V. M. & McCormick, J. J. Mammalian expression vectors with modulatable promoters and two multiple cloning sites. *Gene.* **119**(2), 155–161 (1992). 1327962 10.1016/0378-1119(92)90267-s

[24] CellProfiler 4: Improvements in speed, utility and usability. *BMC Bioinform*. 10.1186/s12859-021-04344-9. 10.1186/s12859-021-04344-9 PMC8431850 34507520

[25] Foxler, D. E. et al. A HIF–LIMD1 negative feedback mechanism mitigates the pro-tumorigenic effects of hypoxia. *EMBO Mol. Med.* **10**(8), e8304 (2018). 29930174 10.15252/emmm.201708304 PMC6079541

[26] Livak, K. J. & Schmittgen, T. D. Analysis of relative gene expression data using real-time quantitative PCR and the 2−ΔΔCT method. *Methods.* **25**(4), 402–408 (2001). 11846609 10.1006/meth.2001.1262

[27] Wigfield, S. M. et al. PDK-1 regulates lactate production in hypoxia and is associated with poor prognosis in head and neck squamous cancer. *Br. J. Cancer.* **98**(12), 1975–1984 (2008). 18542064 10.1038/sj.bjc.6604356 PMC2441961

[28] Coetzee, A. S. et al. Nuclear FGFR1 promotes pancreatic stellate cell-driven invasion through up-regulation of Neuregulin 1. *Oncogene.* **42**(7), 491–500 (2023). 36357571 10.1038/s41388-022-02513-5 PMC9918430

[29] Visualizing the Performance of Scoring Classifiers. https://ipa-tys.github.io/ROCR/.

[30] PanCanAtlas Publications | NCI Genomic Data Commons. https://gdc.cancer.gov/about-data/publications/pancanatlas.

[31] Buffa, F. M., Harris, A. L., West, C. M. & Miller, C. J. Large meta-analysis of multiple cancers reveals a common, compact and highly prognostic hypoxia metagene. *Br. J. Cancer.* **102**(2), 428–435 (2010). 20087356 10.1038/sj.bjc.6605450 PMC2816644

[32] Winter, S. C. et al. Relation of a hypoxia metagene derived from head and neck cancer to prognosis of multiple cancers. *Cancer Res.* **67**(7), 3441–3449 (2007). 17409455 10.1158/0008-5472.CAN-06-3322

[33] Murphy, D. A. & Courtneidge, S. A. The ‘ins’ and ‘outs’ of podosomes and invadopodia: Characteristics, formation and function. *Nat. Rev. Mol. Cell Biol.* **12**(7), 413–426 (2011). 21697900 10.1038/nrm3141 PMC3423958

[34] Ezrin interacts with cortactin to form podosomal rosettes in pancreatic cancer cells. PubMed NCBI. https://www.ncbi.nlm.nih.gov/pubmed/18852256. 10.1136/gut.2008.159871 18852256

[35] Bachem, M. G. et al. Pancreatic carcinoma cells induce fibrosis by stimulating proliferation and matrix synthesis of stellate cells. *Gastroenterology.* **128**(4), 907–921 (2005). 15825074 10.1053/j.gastro.2004.12.036

[36] Gaggioli, C. et al. Fibroblast-led collective invasion of carcinoma cells with differing roles for RhoGTPases in leading and following cells. *Nat. Cell Biol.* **9**(12), 1392–1400 (2007). 18037882 10.1038/ncb1658

[37] Kleinman, H. K. & Martin, G. R. Matrigel: Basement membrane matrix with biological activity. *Semin. Cancer Biol.* **15**(5), 378–386 (2005). 15975825 10.1016/j.semcancer.2005.05.004

[38] Foty, R. a simple hanging drop cell culture protocol for generation of 3D spheroids. *J. Vis. Exp. JoVE.* **51**, 2720 (2011). 10.3791/2720 PMC3197119 21587162

[39] AHNAK2 is a potential prognostic biomarker in patients with PDAC. https://www.ncbi.nlm.nih.gov/pmc/articles/PMC5458247/. 10.18632/oncotarget.15990 PMC5458247 28423668

[40] Zardab, M., Stasinos, K., Grose, R. P. & Kocher, H. M. The obscure potential of AHNAK2. *Cancers.* **14**(3), 528 (2022). 35158796 10.3390/cancers14030528 PMC8833689

[41] Liu, G., Guo, Z., Zhang, Q., Liu, Z. & Zhu, D. AHNAK2 promotes migration, invasion, and epithelial-mesenchymal transition in lung adenocarcinoma cells via the TGF-β/Smad3 pathway. *OncoTargets Ther.* **13**, 12893–12903 (2020). 10.2147/OTT.S281517 PMC7754667 33363388

[42] Yin, M., Ma, W. & An, L. Cortactin in cancer cell migration and invasion. *Oncotarget.* **8**(50), 88232–88243 (2017). 29152154 10.18632/oncotarget.21088 PMC5675706

[43] Song, Y. et al. Ezrin mediates invasion and metastasis in tumorigenesis: A review. *Front. Cell Dev Biol.*10.3389/fcell.2020.588801 (2020). 33240887 10.3389/fcell.2020.588801 PMC7683424

[44] Martinez, J. et al. Microtubule-associated protein 1 light chain 3 alpha (LC3)-associated phagocytosis is required for the efficient clearance of dead cells. *Proc. Natl. Acad. Sci. USA.* **108**(42), 17396–17401 (2011). 21969579 10.1073/pnas.1113421108 PMC3198353

[45] Huynh, K. K. et al. LAMP proteins are required for fusion of lysosomes with phagosomes. *EMBO J.* **26**(2), 313–324 (2007). 17245426 10.1038/sj.emboj.7601511 PMC1783450

[46] Yang, S. et al. Pancreatic cancers require autophagy for tumor growth. *Genes Dev.* **25**(7), 717–729 (2011). 21406549 10.1101/gad.2016111 PMC3070934

[47] Chiou, S. H. et al. BLIMP1 induces transient metastatic heterogeneity in pancreatic cancer. *Cancer Discov.* **7**(10), 1184–1199 (2017). 28790031 10.1158/2159-8290.CD-17-0250 PMC5628145

[48] Zheng, L., Li, S., Zheng, X., Guo, R. & Qu, W. AHNAK2 is a novel prognostic marker and correlates with immune infiltration in papillary thyroid cancer: Evidence from integrated analysis. *Int. Immunopharmacol.* **1**(90), 107185 (2021). 10.1016/j.intimp.2020.107185 33218938

[49] Kyriazis, A. A., Kyriazis, A. P., Sternberg, C. N., Sloane, N. H. & Loveless, J. D. Morphological, biological, biochemical, and karyotypic characteristics of human pancreatic ductal adenocarcinoma Capan-2 in tissue culture and the nude mouse. *Cancer Res.* **46**(11), 5810–5815 (1986). 3019537

[50] Goonetilleke, K. S. & Siriwardena, A. K. Systematic review of carbohydrate antigen (CA 19–9) as a biochemical marker in the diagnosis of pancreatic cancer. *Eur. J. Surg. Oncol. EJSO.* **33**(3), 266–270 (2007). 17097848 10.1016/j.ejso.2006.10.004

[51] Satake, K. & Takeuchi, T. Comparison of CA19-9 with other tumor markers in the diagnosis of cancer of the pancreas. *Pancreas.* **9**(6), 720 (1994). 7846015 10.1097/00006676-199411000-00008

[52] Silva, T. A. et al. AHNAK enables mammary carcinoma cells to produce extracellular vesicles that increase neighboring fibroblast cell motility. *Oncotarget.* **7**(31), 49998–50016 (2016). 27374178 10.18632/oncotarget.10307 PMC5226564

