# Supplementary material for: AHNAK2: a potential diagnostic biomarker for pancreatic cancer related to cellular motility
Source: Sci Rep. 2025 Jan 23;15:2934. doi: 10.1038/s41598-025-87337-5 (PMC11757713; doi:10.1038/s41598-025-87337-5)
Supplement: Supplementary file 1 — Supplementary Information. [file 41598_2025_87337_MOESM1_ESM.docx]

## Supplementary

| **Antibody** | **Dilution** | **Primary Incubation** | **Secondary Antibody** | **Secondary Incubation** |
| --- | --- | --- | --- | --- |
| anti-AHNAK2 (HPA000878, Sigma-Aldrich^®^, USA) | 1:250 | 60 minutes | Biotinylated secondary goat anti-rabbit antibody (1858415, Pierce^®^, UK) (1:200) | 40 minutes |
| Polyclonal IgG rabbit isotype (ab172730, Abcam^®^, UK) | 1:500 | 60 minutes | Biotinylated secondary goat anti-rabbit antibody (1858415, Pierce^®^, UK) (1:200) | 40 minutes |

**Supplementary Table 1** **– List of antibodies**
A list of antibodies used during the IHC experiments, incubation was at room temperature (RT).

| **Antibody** | **Dilution** | **Primary Incubation** | **Secondary Antibody** | **Secondary Incubation** |
| --- | --- | --- | --- | --- |
| anti-AHNAK2 (HPA000878, Sigma-Aldrich^®^, USA) | 1:250 | 60 minutes | Alexa-Fluor goat anti-rabbit 488 (A11034)(1:500) | 40 minutes |
| Polyclonal IgG rabbit isotype (ab172730, Abcam^®^, UK) | 1:500 | 60 minutes | Alexa-Fluor goat anti-rabbit 488 (A11034)(1:500) | 40 minutes |
| anti-E-cadherin antibody (ab1416, Abcam^®^, UK) | 1:200 | 60 minutes | Alexa-Fluor anti-mouse 546 (A11081)(1:500) | 40 minutes |
| anti-Vimentin antibody (M0725, Dako^®^, USA) | 1:100 | 60 minutes | Alexa-Fluor anti-mouse 546 (A11081)(1:500) | 40 minutes |
| anti-Ezrin (610603, BD Biosciences^®^, USA) | 1:250 | 60 minutes | Alexa-Fluor anti-mouse 546 (A11081)(1:500) | 40 minutes |
| anti-Cortactin (05-180, Upstate^®^, USA) | 1:500 | 60 minutes | Alexa-Fluor anti-mouse 546 (A11081)(1:500) | 40 minutes |
| Polyclonal IgG mouse (X0931, Dako^®^, USA) | Variable | 60 minutes | Alexa-Fluor anti-mouse 546 (A11081) | 40 minutes |

**Supplementary Table 2** **– List of antibodies**
A list of antibodies used during the Immunofluorescence experiments, incubation was at room temperature (RT).

| **Cell line** | **Source** | **Differentiation** | **Doubling time** | **KRAS** | **TP53** | ***SMAD4*** | **AHNAK2 Protein expression** |
| --- | --- | --- | --- | --- | --- | --- | --- |
| MIA PaCa-2 | Primary tumour | Poorly differentiated | 40 hours | G12C | R248W | WT | High, cytoplasmic and found at cellular protrusions. |
| Capan-2 | Primary tumour | Well differentiated | 96 hours | G12V | T125T* | WT | Moderate, cytoplasmic, found at cell junction and cellular protrusions. |
| COLO357 | Lymph node metastasis | Poorly differentiated | 21 hours | G12D | WT | Homozygous deletion | Moderate, cytoplasmic, vesicular pattern but also found at cellular protrusions. |
| Capan-1 | Liver metastasis | Well differentiated | 41 hours | G12V | A159V | Mutation causing loss of expression | Low cytoplasmic, mostly vesicular pattern and at cell protrusions. |
| RWP-1 | Liver metastasis | Well differentiated | 45 hours | G12A | R175H | Unknown | Moderate cytoplasmic and cell membrane. |
| BXPC-3 | Primary tumour | Moderately differentiated | 48 hours | WT | Y220C | Homozygous deletion | Low to no expression, no particular pattern of expression. |

**Supplementary Table 3 – Cell line characteristics**A summary of the main characteristics of six cell lines with the three cell lines chosen for further experimentation^1–5^. ***Silent mutation but impairs splicing^6^.

| **Gene Set Ontology (Positive correlation)** | **# Genes in Overlap (k)** | **k/K** | **p-value** |
| --- | --- | --- | --- |
| GO_PHOSPHOLIPASE_A2_INHIBITOR_ACTIVITY | 25 | 0.0453 | 4.75E-22 |
| GO_HEMIDESMOSOME_ASSEMBLY | 32 | 0.0251 | 2.10E-20 |
| GO_MEMBRANE_RAFT_ASSEMBLY | 20 | 0.0489 | 2.07E-18 |
| GO_PROTEIN_COMPLEX_INVOLVED_IN_CELL_ADHESION | 21 | 0.043 | 3.66E-18 |
| GO_CELL_SUBSTRATE_JUNCTION_ASSEMBLY | 28 | 0.0198 | 3.07E-15 |
| GO_CELL_ADHESION_MEDIATED_BY_INTEGRIN | 15 | 0.0463 | 1.06E-13 |
| GO_BASEMENT_MEMBRANE | 13 | 0.0544 | 6.62E-13 |
| GO_FORMATION_OF_PRIMARY_GERM_LAYER | 6 | 0.5 | 7.11E-13 |
| GO_CELL_JUNCTION_ASSEMBLY | 16 | 0.0348 | 1.21E-12 |
| GO_CELL_SUBSTRATE_JUNCTION | 15 | 0.0362 | 3.59E-12 |

**Supplementary Table 4 – Gene set ontology groups of positively correlated genes**
Top ten gene set ontology groups provided by gene set enrichment analysis of the top fifty positively correlated genes to AHNAK2. (Spearman correlation) from the TCGA database. k/K value provides the ratio of genes correlated to the number of genes in the gene set ontology. Most of the genes are related to cell junction formation and communication between the cell and extracellular matrices. Important genes of note include Annexin A2 (cell motility), Zinc Finger Proteins, various Cadherins (cell to cell adhesion), Cortactin, Ezrin and INPP4B (involved in the phosphatidylinositol signalling pathway).

| **Gene Set Ontology (Negative correlation)** | **# Genes in Overlap (k)** | **k/K** | **p-value** |
| --- | --- | --- | --- |
| GO_COFACTOR_METABOLIC_PROCESS | 0.0203 | 2.53E-06 | 2.53E-02 |
| GO_CYTOPLASMIC_VESICLE_PART | 0.0101 | 7.06E-06 | 3.53E-02 |

**Supplementary Table 5 - Gene set ontology groups of negatively correlated genes**
Top ten gene set ontology groups provided by gene set enrichment analysis of the top fifty genes negatively correlated to AHNAK2 Only two gene set ontology groups were generated from the list of the top fifty negatively correlated genes from the TCGA database. Some genes of note include Acetyl-CoA carboxylase Beta (ACACB), acyl-CoA synthase (ACSL6), vascular endothelial growth factor D (VEGFD) and cell death inducing P53 target 1 (CDIP1).

| **MIA PaCa-2** | **Substrate experiments** | | | **Normoxia versus hypoxia** | | |
| --- | --- | --- | --- | --- | --- | --- |
|  | **Fibronectin** | **Collagen** | **Matrigel** | **Hypoxia vs Normoxia** | **AHNAK2 siRNA vs NT in Normoxia** | **AHNAK2 siRNA vs NT in Hypoxia** |
| ***AHNAK2* mRNA expression** | NS | NS | NS | Increase** | Decrease**** | Decrease**** |
| **AHNAK2 protein expression** | More than Matrigel** | NS | Less than Fibronectin** | Increase**** | Decrease**** | Decrease**** |
| **Cortactin protein expression** | NS | NS | NS | NS | NS | Increase**** |
| **Ezrin protein expression** | NS | NS | NS | Increase*** | Increase**** | NS |
| **AHNAK2 Cortactin co-localisation** | More than Matrigel* | NS | Less than Fibronectin* | Increase* | NA | NA |
| **AHNAK2 Ezrin co-localisation** | NS | NS | NS | Decrease* | NA | NA |
| **Cell Eccentricity** | NA | NA | NA | NA | Increase**** | Increase**** |
| **Cell area** | NA | NA | NA | NA | Increase**** | Increase** |
| **Cell length of axis** | NA | NA | NA | NA | Increase**** | Increase**** |

**Supplementary Table 6** **– A summary of results for experiments on the MIA PaCa-2 cell line**
A summary of the results of the various experiments in different conditions of the MIAPaCa-2 cell line. (* = p<0.05, ** = p<0.01, ***=p<0.001, ****=p<0.0001, NS = non-significant). NA is not applicable. The appropriate test is described in the relevant figure for each experiment.

| **COLO357** | **Substrate experiments** | | | **Normoxia versus hypoxia** | | |
| --- | --- | --- | --- | --- | --- | --- |
|  | **Fibronectin** | **Collagen** | **Matrigel** | **Hypoxia vs Normoxia** | **AHNAK2 siRNA vs NT in Normoxia** | **AHNAK2 siRNA vs NT in Hypoxia** |
| ***AHNAK2* mRNA expression** | NS | NS | NS | Increase* | Decrease**** | Decrease**** |
| **AHNAK2 protein expression** | More than Matrigel*** | More than Matrigel**** | Less than Collagen**** Less than Fibronectin*** | Increase**** | Decrease**** | Decrease**** |
| **Cortactin protein expression** | More than Matrigel* | More than Matrigel** | Less than Collagen** Less than Fibronectin* | Decrease**** | Increase**** | Decrease**** |
| **Ezrin protein expression** | Less than Collagen**** | More than Fibronectin**** | Less than Collagen** | Decrease**** | Increase** | Decrease**** |
| **AHNAK2 Cortactin co-localisation** | NS | NS | NS | NS | NA | NA |
| **AHNAK2 Ezrin co-localisation** | NS | NS | NS | NS | NA | NA |
| **Cell Eccentricity** | NA | NA | NA | NA | NS | NS |
| **Cell area** | NA | NA | NA | NA | Decrease**** | Decrease**** |
| **Cell length of axis** | NA | NA | NA | NA | Decrease**** | Decrease**** |

**Supplementary Table 7** **– A summary of results for experiments on the COLO357 cell line**
A summary of the results of the various experiments in different conditions of the COLO357 cell line. (* = p<0.05, ** = p<0.01, ***=p<0.001, ****=p<0.0001, NS = non-significant). NA is not applicable. The appropriate test is described in the relevant figure for each experiment.

| **Capan-2** | **Substrate experiments** | | | **Normoxia versus hypoxia** | | |
| --- | --- | --- | --- | --- | --- | --- |
|  | **Fibronectin** | **Collagen** | **Matrigel** | **Hypoxia vs Normoxia** | **AHNAK2 siRNA vs NT in Normoxia** | **AHNAK2 siRNA vs NT in Hypoxia** |
| ***AHNAK2* mRNA expression** | NS | NS | NS | Decrease** | Decrease**** | Decrease**** |
| **AHNAK2 protein expression** | NS | NS | NS | Increase**** | Decrease**** | Decrease**** |
| **Cortactin protein expression** | Less than Matrigel* More than Collagen**** | Less than Fibronectin**** Less than Matrigel**** | More than Collagen**** | Increase**** | Increase**** | Decrease**** |
| **Ezrin protein expression** | More than Matrigel**** | More than Matrigel** | Less than Fibronectin**** Less than Collagen** | Decrease** | Decrease**** | NS |
| **AHNAK2 Cortactin co-localisation** | More than Matrigel* More than Collagen** | Less than Fibronectin** | Less than Fibronectin* | Increase** | NA | NA |
| **AHNAK2 Ezrin co-localisation** | NS | NS | NS | NS | NA | NA |
| **Cell Eccentricity** | NA | NA | NA | NA | NS | NS |
| **Cell area** | NA | NA | NA | NA | Increase**** | NS |
| **Cell length of axis** | NA | NA | NA | NA | Increase*** | Decrease** |

**Supplementary Table** **8** **– A summary of results for experiments on the Capan-2 cell line**
A summary of the results of the various experiments in different conditions of the Capan-2 cell line. (* = p<0.05, ** = p<0.01, ***=p<0.001, ****=p<0.0001, NS = non-significant). NA is not applicable. The appropriate test is described in the relevant figure for each experiment.

| **Characteristics** | **PDAC (n=30)** |
| --- | --- |
| **Age** |  |
| Median | 69.00 |
| Range | 45.00 - 87.00 |
| Q1-Q3 | 62.25, 73.75 |
| Unknown | 0 |
| **Gender** |  |
| Female | 15 (50.0%) |
| Male | 15 (50.0%) |
| Unknown | 0 |
| **T stage** |  |
| 1 | 1 (3.7%) |
| 2 | 5 (18.5%) |
| 3 | 14 (51.9%) |
| 4 | 7 (25.9%) |
| Unknown | 3 |
| **N stage** |  |
| 0 | 13 (48.1%) |
| 1 | 12 (44.4%) |
| Unknown | 5 |
| **M stage** |  |
| 0 | 16 (59.3%) |
| 1 | 11 (40.7%) |
| Unknown | 3 |
| **Stage** |  |
| IA | 1 (3.7%) |
| IIA | 5 (18.5%) |
| IIB | 9 (33.3%) |
| III | 1 (3.7%) |
| IV | 11 (40.7%) |
| Unknown | 3 |
| **Staging** |  |
| Stage 2 or less | 15 (55.6%) |
| Stage 3 or more | 12 (44.4%) |
| Unknown | 3 |
| **Resection status** |  |
| R0 | 10 (33.3%) |
| R1 | 4 (13.3%) |
| Unknown | 16 (53.3%) |
| Unknown | 0 |
| **Invasion** |  |
| Both | 11 (36.7%) |
| None | 1 (3.3%) |
| Peri-neural | 4 (13.3%) |
| Unknown | 14 (46.7%) |
| Unknown | 0 |
| **Treatment** |  |
| Chemotherapy | 19 (65.5%) |
| No | 10 (34.5%) |
| Unknown | 1 |
| **CA 19-9 levels** |  |
| Median | 1065.00 |
| Range | 0.00 - 22376.00 |
| Q1-Q3 | 98.50, 5635.50 |
| Unknown | 15 |
| **Red blood cell count** |  |
| Median | 4.04 |
| Range | 3.08 - 5.16 |
| Q1-Q3 | 3.53, 4.32 |
| Unknown | 3 |
| **Sodium levels** |  |
| Median | 138.00 |
| Range | 126.00 - 146.00 |
| Q1-Q3 | 132.00, 140.00 |
| Unknown | 2 |
| **Bilirubin levels** |  |
| Median | 11.00 |
| Range | 5.00 - 257.00 |
| Q1-Q3 | 6.50, 27.50 |
| Unknown | 3 |
| **AHNAK2 levels** |  |
| Median | 565.98 |
| Range | 66.61 - 1364.31 |
| Q1-Q3 | 457.03, 721.65 |
| Unknown | 0 |
| **Median Tumour expression of AHNAK2** |  |
| Median | 145.78 |
| Range | 102.61 - 196.92 |
| Q1-Q3 | 132.40, 164.36 |
| Unknown | 16 |
| **Median Nerve cell expression of AHNAK2** |  |
| Median | 102.05 |
| Range | 92.18 - 170.51 |
| Q1-Q3 | 98.84, 122.88 |
| Unknown | 16 |
| **Median Stromal expression of AHNAK2** |  |
| Median | 93.01 |
| Range | 58.77 - 107.14 |
| Q1-Q3 | 77.65, 98.62 |
| Unknown | 16 |
| **Median of all tissue expression of AHNAK2** |  |
| Median | 126.76 |
| Range | 85.97 - 196.49 |
| Q1-Q3 | 111.67, 149.93 |
| Unknown | 16 |

**Supplementary Table 8– PDAC patient features**
A table of the clinicopathological features, biochemical results and tissue expression of AHNAK2 of the PDAC patients included in the study (n=30).


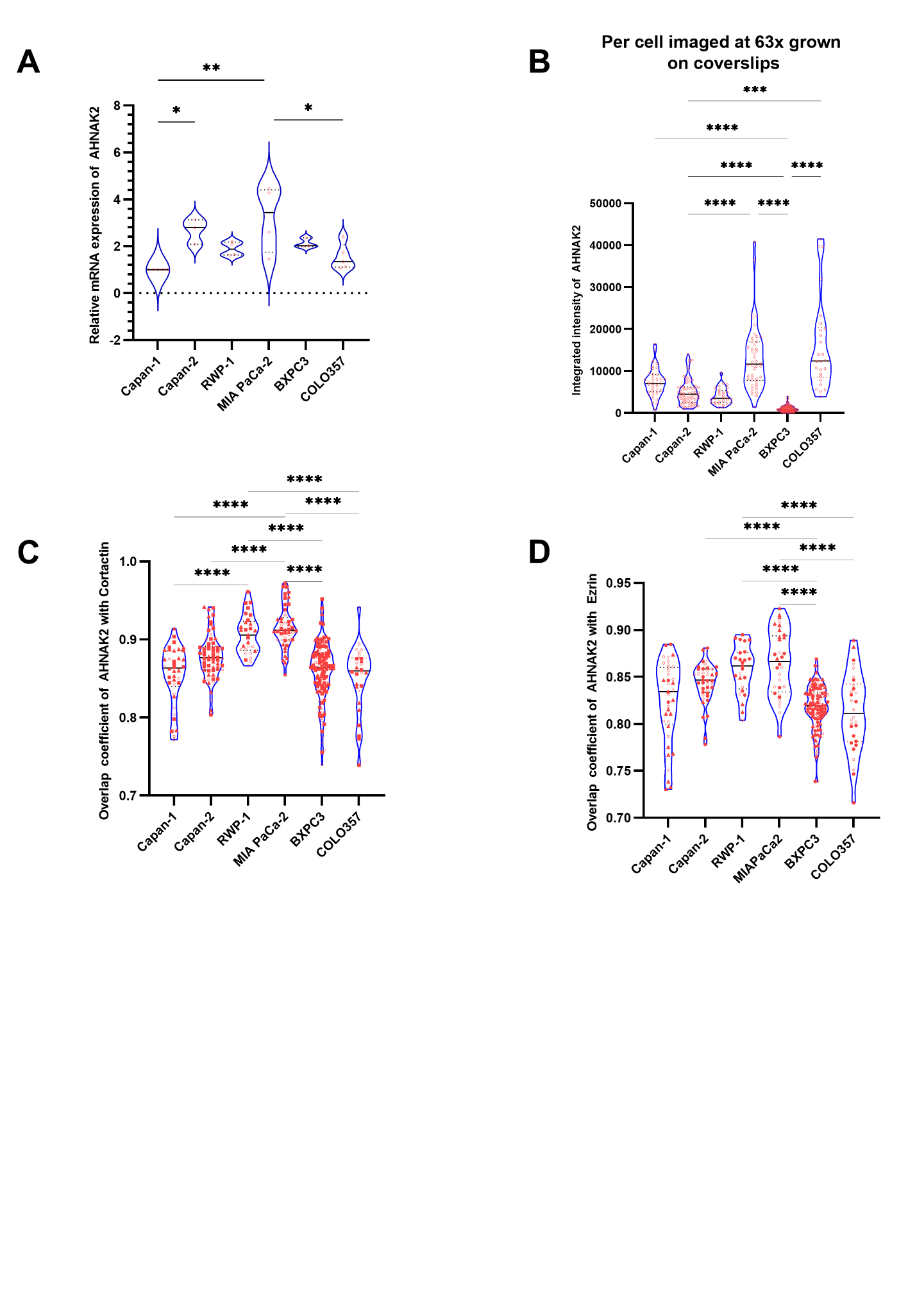
**Supplementary Figure 1 - AHNAK2, Ezrin and Cortactin expression in pancreatic cancer cell lines
A.** AHANK2 mRNA expression in six representative pancreatic cancer cell lines with Capan-1 acting as a reference (n=5). **B.** AHNAK2 protein expression in six representative pancreatic cancer cell lines by immunostaining (CellProfiler), each symbol signifies a cell with each biological repeat presented with a different symbol (n=3). **C, D.** Overlap coefficient of AHNAK2 with Cortactin and Ezrin, each symbol signifies a cell with each biological repeat presented with a different symbol (n=3). Kruskal-Wallis with post-hoc Dunn test for multiple comparisons. *, p<0.05; **, p<0.01; ***, p<0.001. ****, p<0.0001.


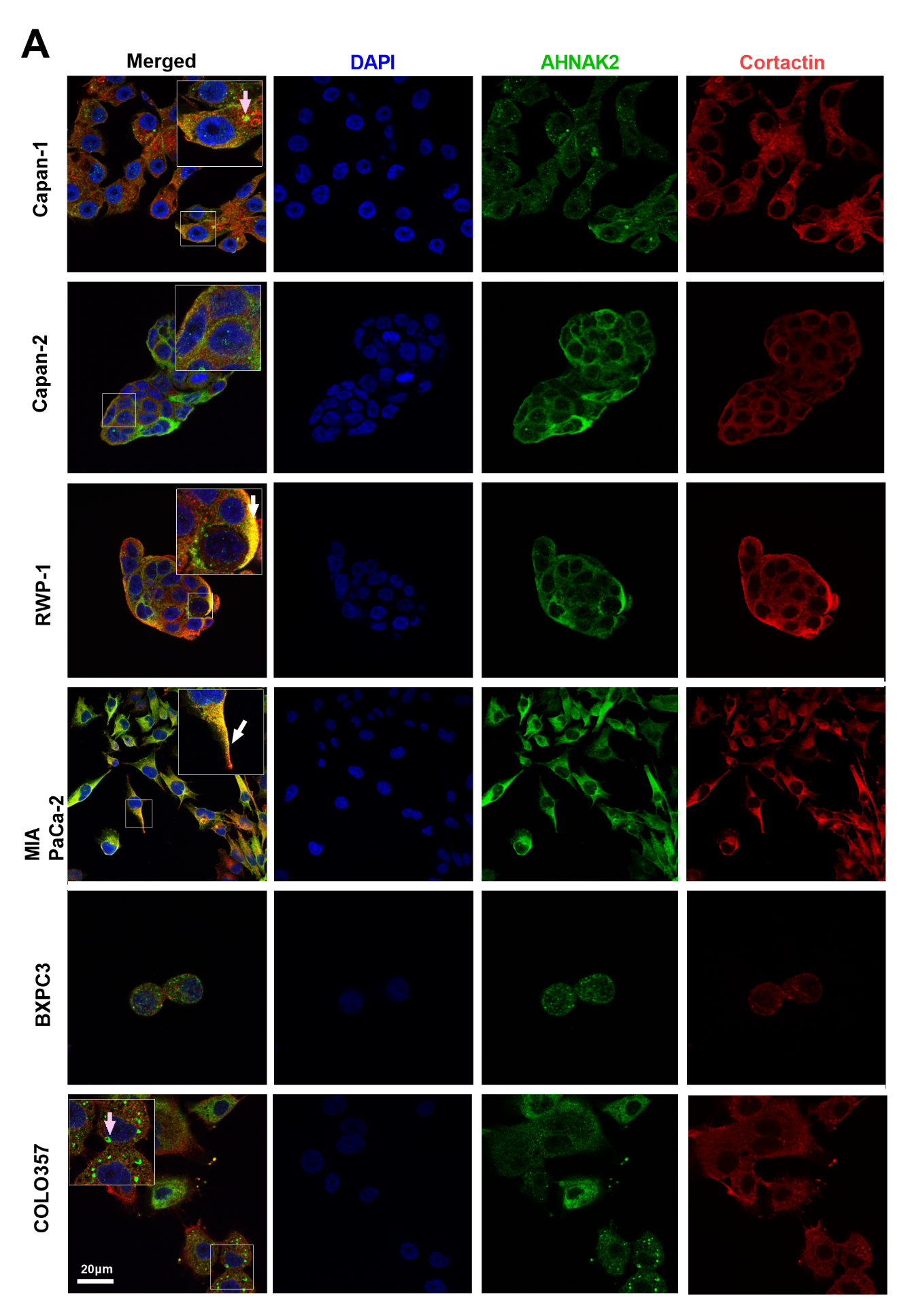
**Supplementary Figure 2 – AHNAK2 and Cortactin expression
A.** Immunofluorescence with AHNAK2 and Cortactin to characterise AHNAK2s involvement in cellular protrusions (white arrows) and vesicles (pink arrows) with insets showing magnification of some cells.


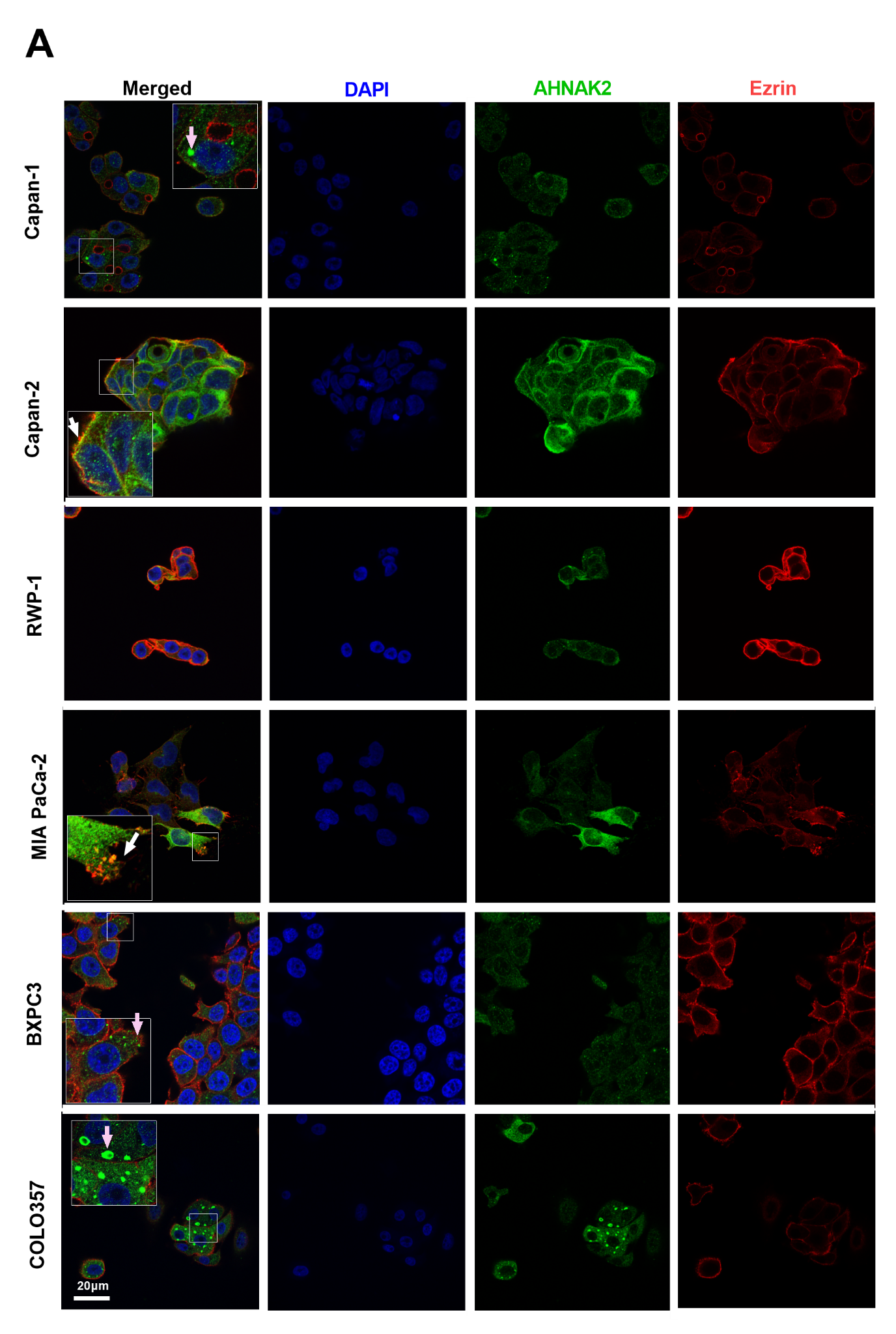
**Supplementary Figure 3 – AHNAK2 and Ezrin expression
A.** Immunofluorescence with AHNAK2 and Ezrin to characterise AHNAK2s involvement in cellular protrusions (white arrows) and vesicles (pink arrows) with insets showing magnification of some cells.


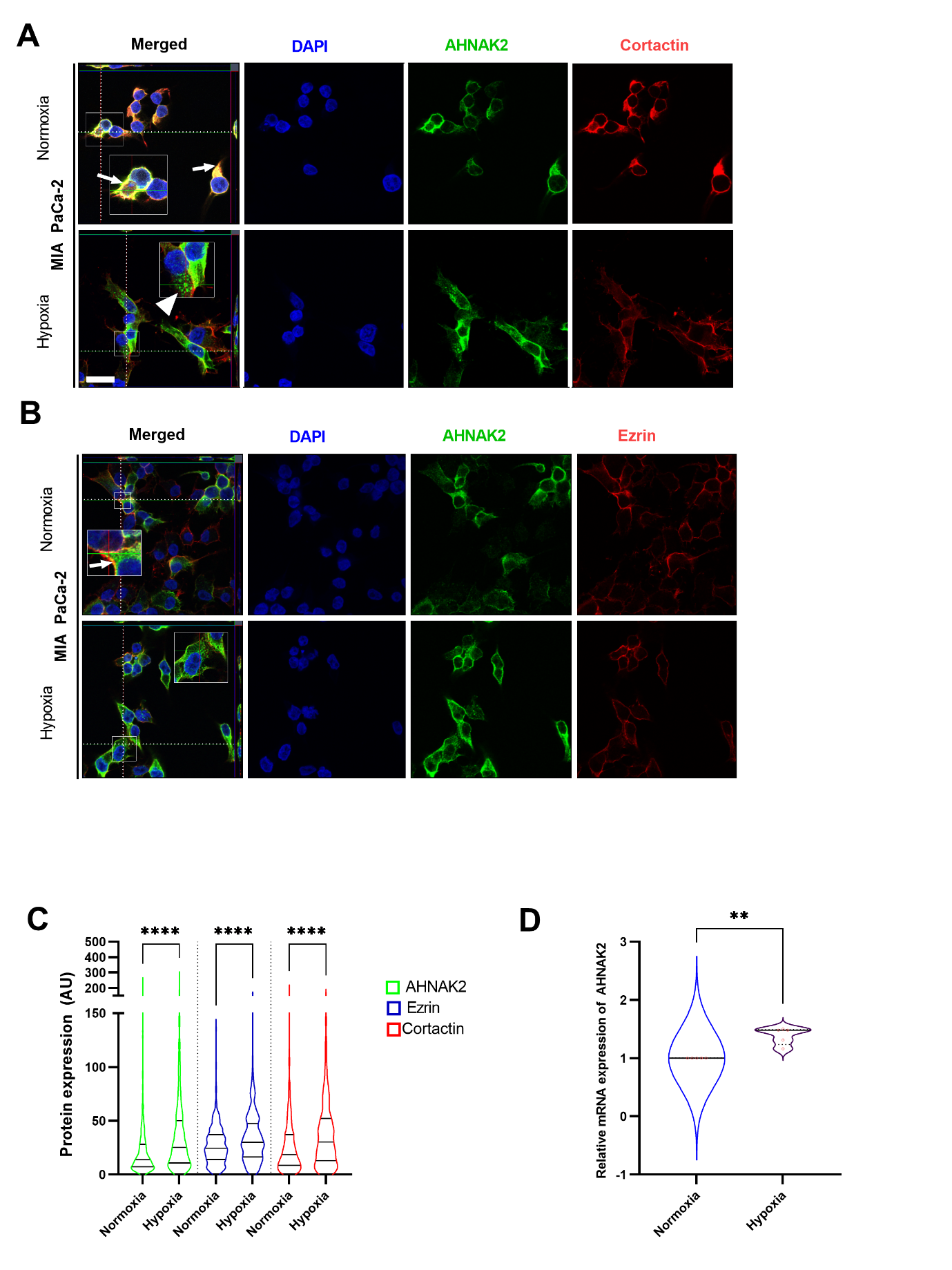


**Supplementary Figure 4 – MIAPaCa-2 grown in normoxic and hypoxic conditions**Immunofluorescence with AHNAK2 and (**A)** Cortactin and (**B)** Ezrin to characterise AHNAK2s involvement in cellular protrusions (white arrows) and vesicles (pink arrows) with inserts showing magnification of some cells. **C.** AHNAK2, Ezrin and Cortactin proteins expression in hypoxia and normoxia as measured by CellProfiler (>1000 cells, (n=5) biological replicates). **D.** AHNAK2 mRNA expression with GAPDH as the house keeping gene in normoxia and RPII in hypoxia (n=5). A two-tailed Mann-Whitney-U test. **, p<0.01; ****, p<0.0001. Scale bar = 20µm.

**
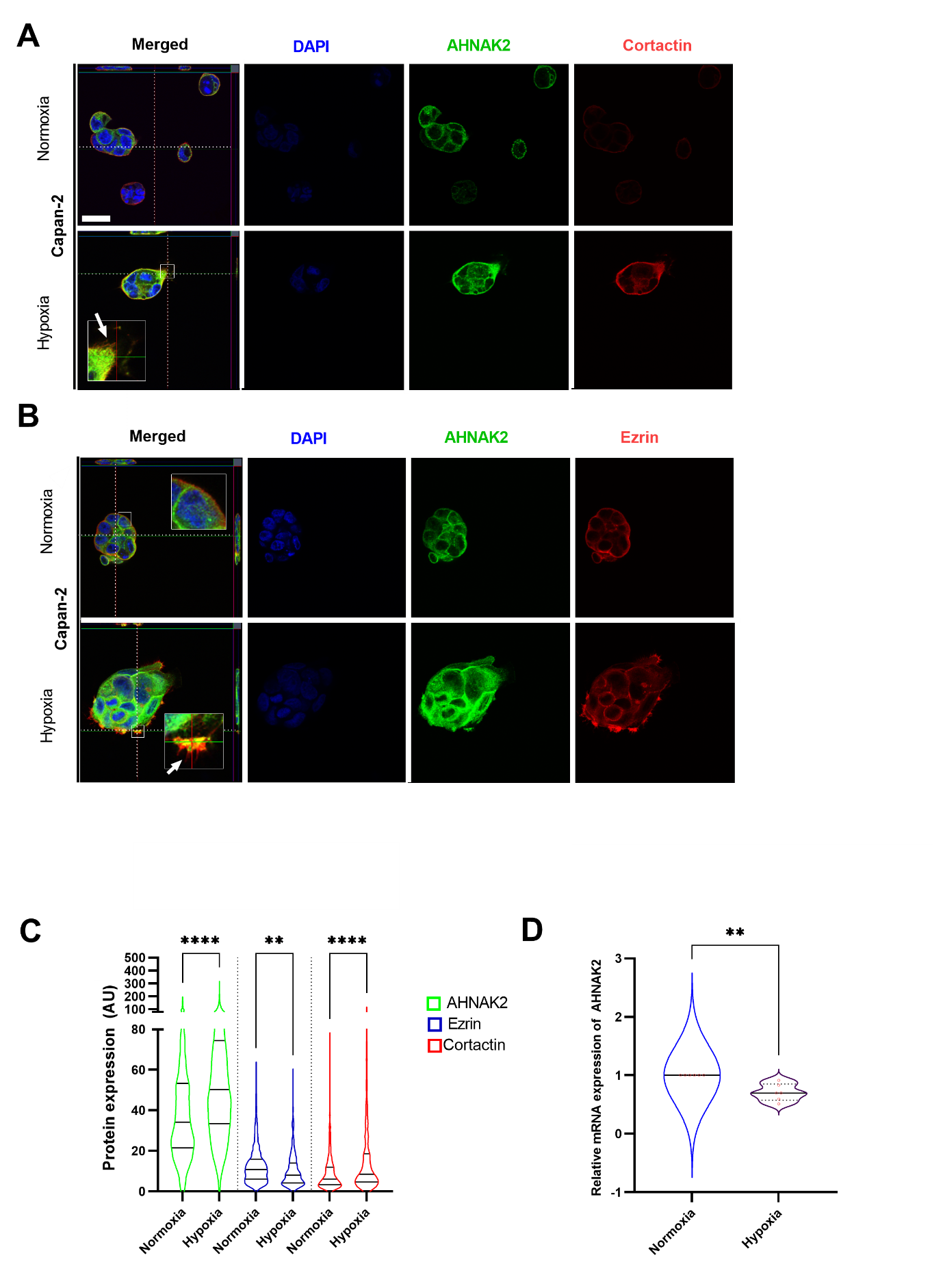
Supplementary Figure 5 – Capan-2 grown in normoxic and hypoxic conditions**Immunofluorescence with AHNAK2 and (**A)** Cortactin and (**B)** Ezrin to characterise AHNAK2s involvement in cellular protrusions (white arrows) with insets showing magnification of some cells. **C.** AHNAK2, Ezrin and Cortactin proteins expression in hypoxia and normoxia as measured by CellProfiler (>1000 cells, 5 biological replicates). **D.** AHNAK2 mRNA expression with GAPDH as the house keeping gene in normoxia and RPII in hypoxia (n=5). A two-tailed Mann-Whitney-U test. **, p<0.01; ****, p<0.0001. Scale bar = 20µm.

**
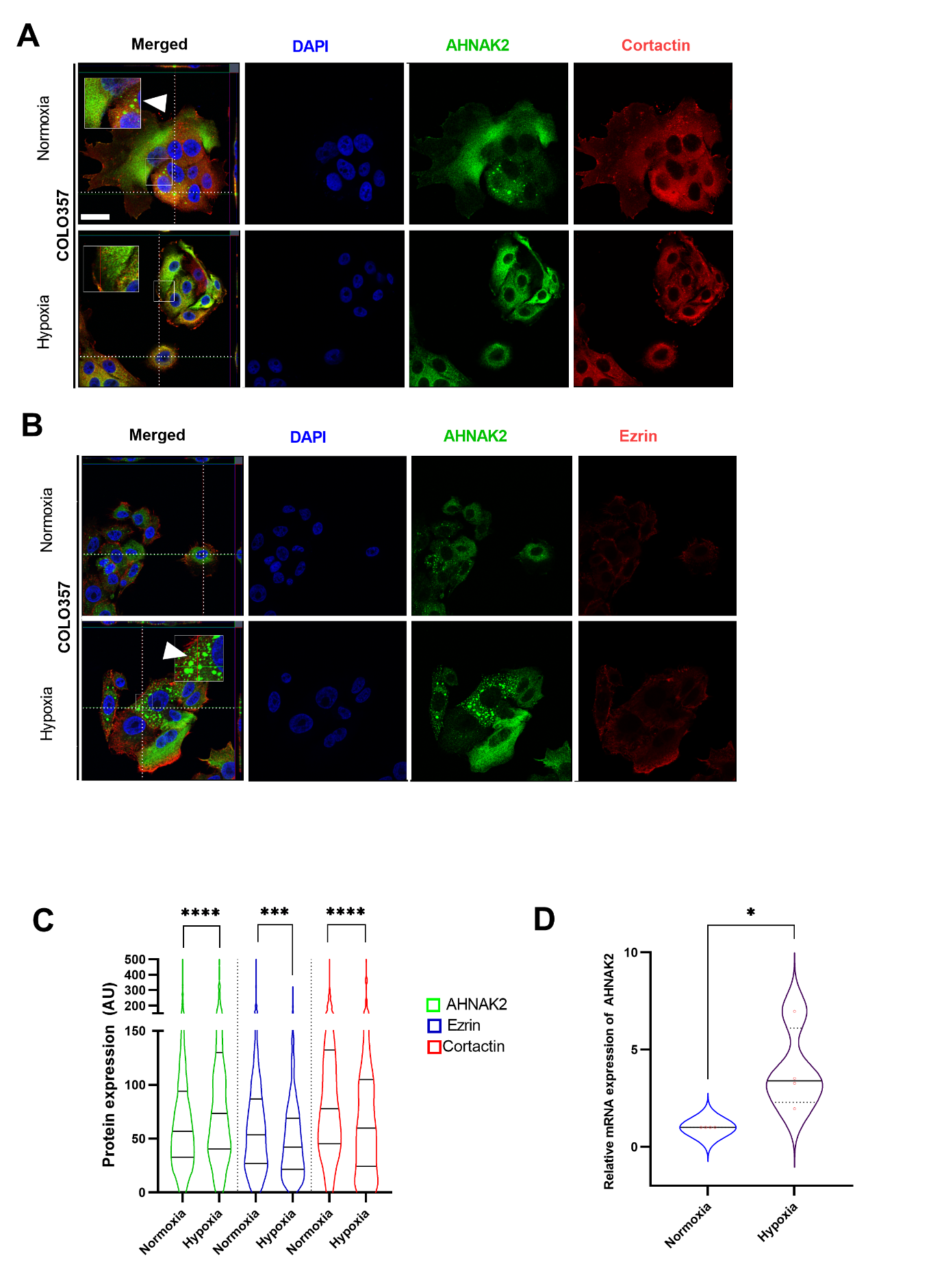
Supplementary Figure 6** **– COLO357 grown in normoxic and hypoxic conditions**
Immunofluorescence with AHNAK2 and (**A)** Cortactin and (**B)** Ezrin to characterise AHNAK2s involvement in cellular protrusions (white arrows) with insets showing magnification of some cells. **C.** AHNAK2, Ezrin and Cortactin proteins expression in hypoxia and normoxia as measured by CellProfiler (>1000 cells, 5 biological replicates). **D.** AHNAK2 mRNA expression with GAPDH as the house keeping gene in normoxia and RPII in hypoxia (n=5). A two-tailed Mann-Whitney-U test. *, p<0.05; **, p<0.01; ***, p<0.001; ****, p<0.0001. Scale bar = 20µm.


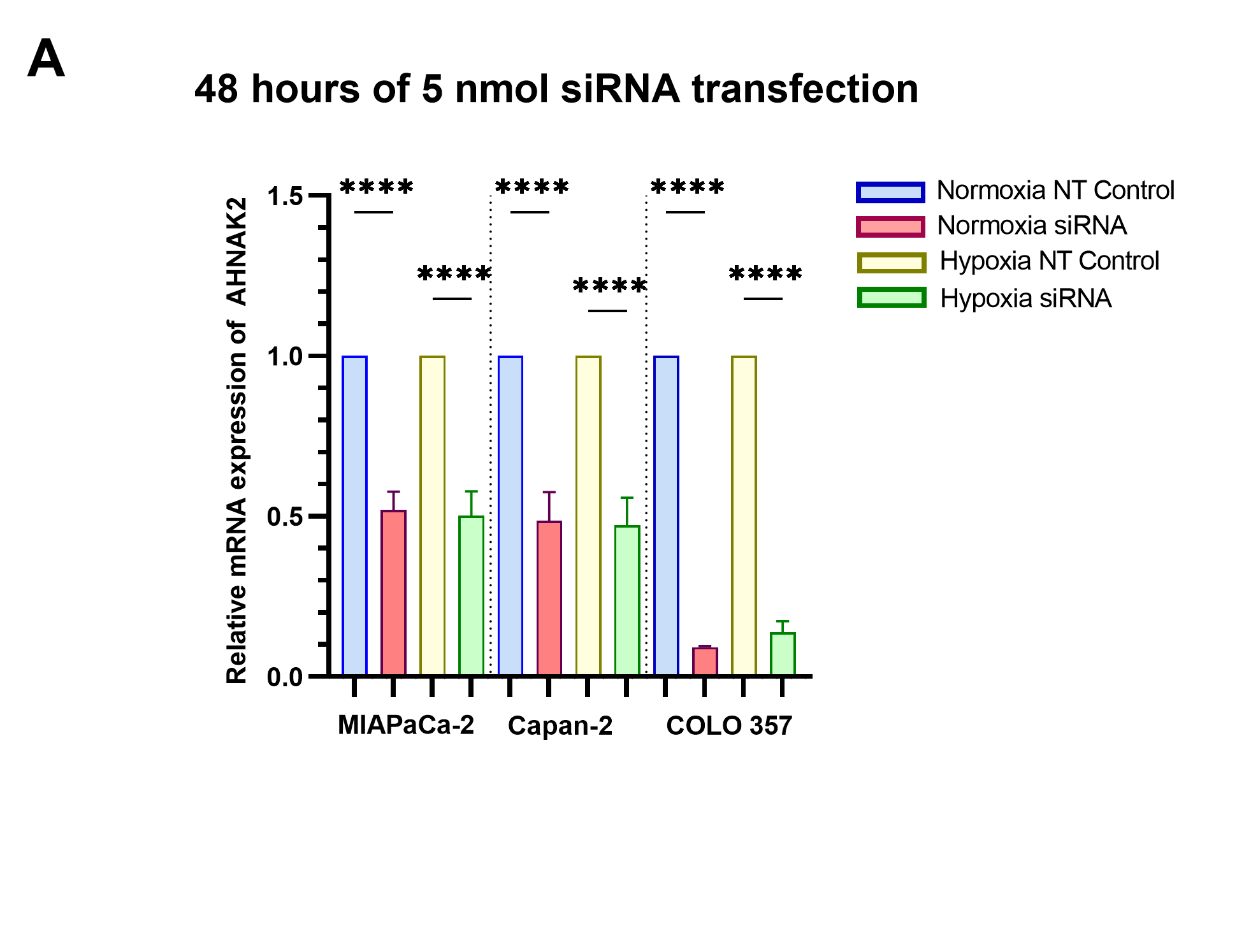
 **Supplementary Figure 7** **– Reduced expression of AHNAK2 in cell lines after AHNAK2 siRNA transfection in Normoxia and Hypoxia**
**A.** Relative mRNA expression of AHNAK2 in all three cell lines in normoxia and hypoxia after transfection with non-targeting control siRNA and AHNAK2 siRNA. ΔΔCT was used for expression with GAPDH as the house keeping gene in normoxia and RPII in hypoxia (n=3). A two-tailed Mann-Whitney-U test. ****, p<0.0001.


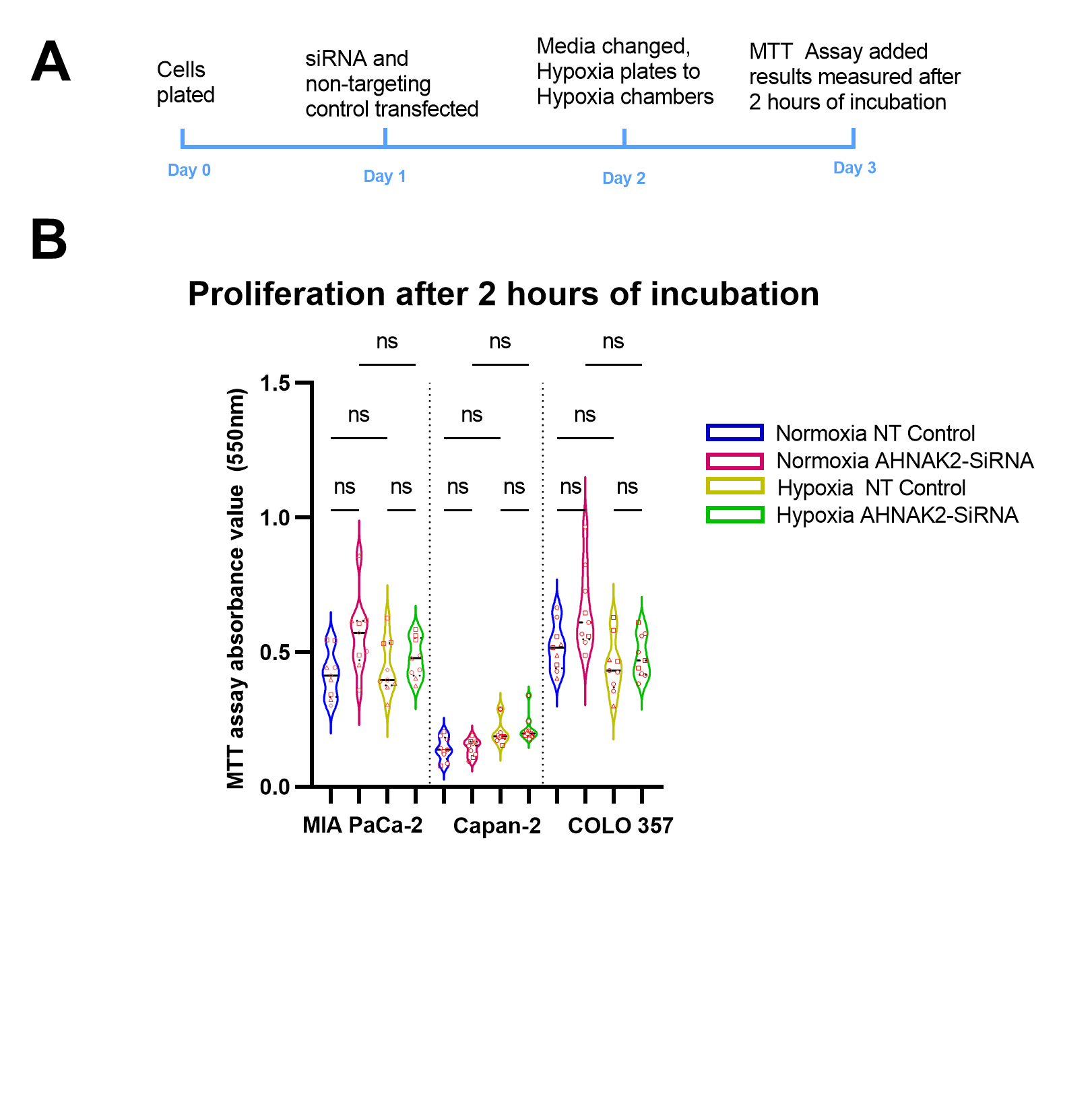


**Supplementary Figure 8 – AHNAK2 silencing and proliferation in normoxia and hypoxia
A.** Experimental schema for measurement of AHNAK2 mRNA (6 well) after siRNA as well effect on proliferation (96well, MTT assay). **B.** MTT Proliferation assay at 48h after transfection and 24h after hypoxia (n=3). Two-tailed Mann-Whitney U tests. ns, not significant.


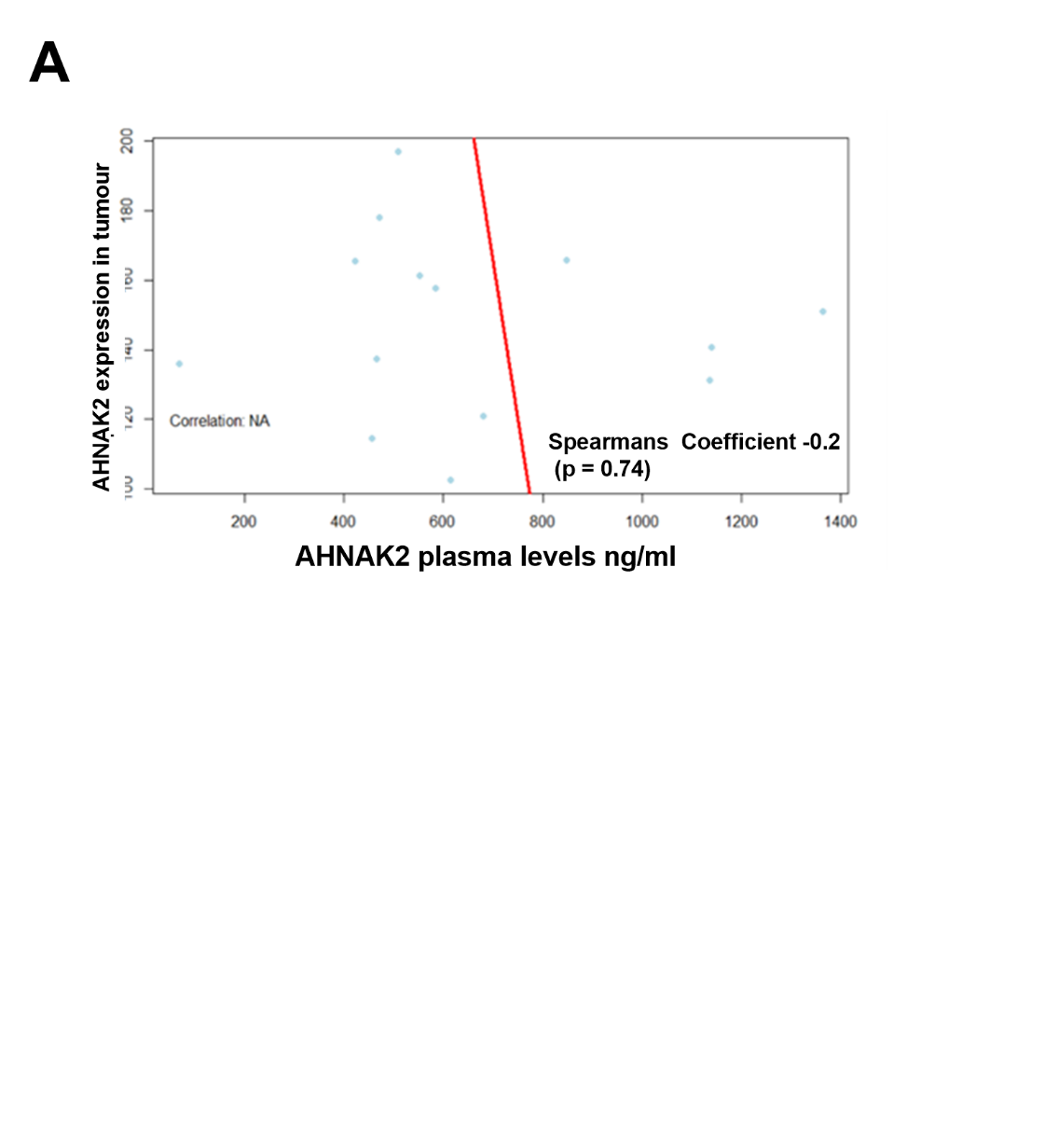


**Supplementary Figure 9 –AHNAK2 expression in tumour and plasma samples of patients with PDAC
A.**  Correlation with AHNAK2 plasma levels and median expression of AHNAK2 in tumours. Log-rank test for Kaplan-Meier curves, Spearman’s test for correlation.


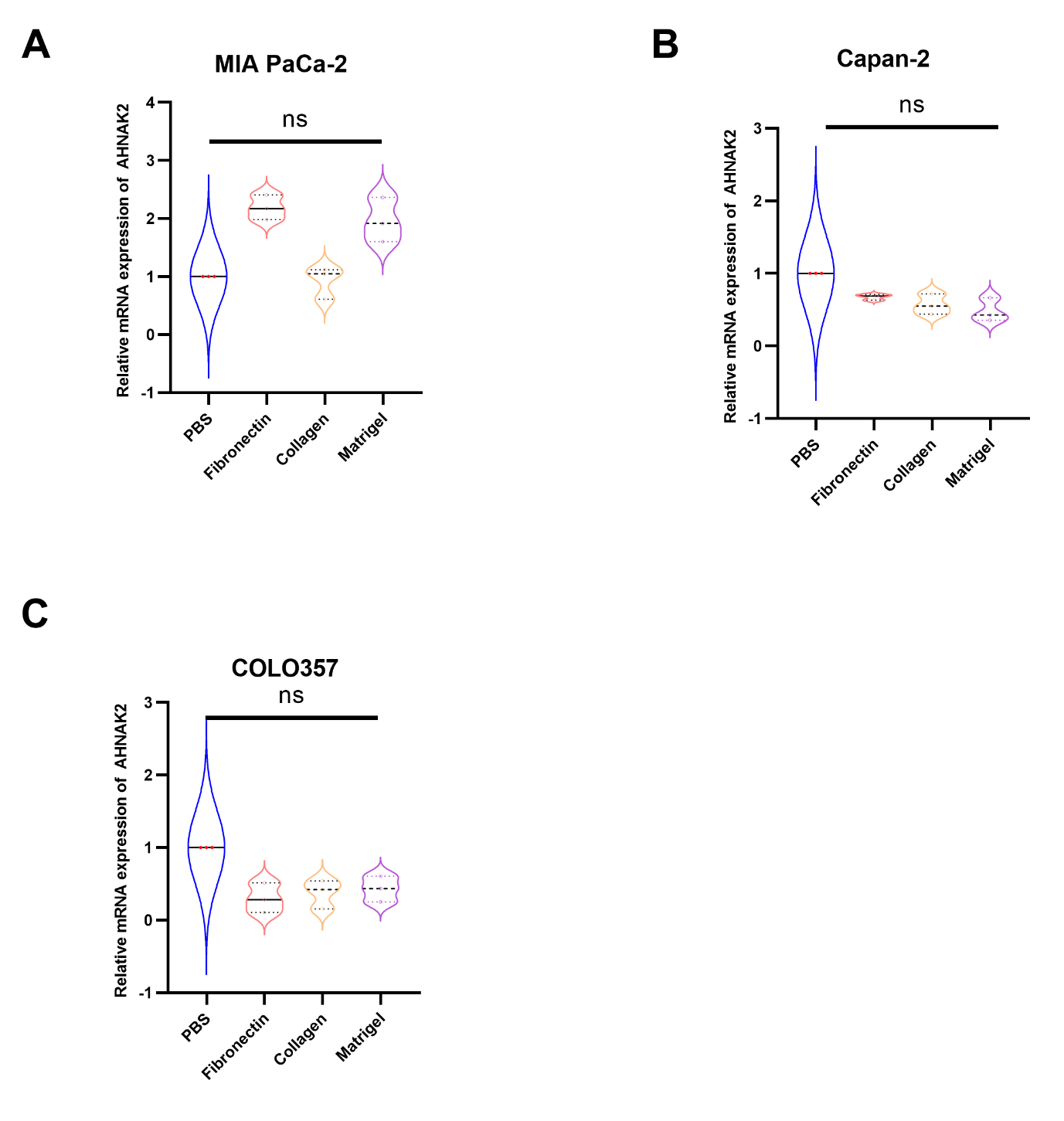


**Supplementary Figure 10 – AHNAK2 mRNA expression is unchanged when cells are grown on different substrates
A.** MIA PaCa-2 **B.** Capan-2 **C.** COLO357 AHNAK2 mRNA expression on different Kruskal-Wallis with post-hoc Dunn test for multiple comparisons. ns: not significant
